# What is Functional Communication? A Theoretical Framework for Real-World Communication Applied to Aphasia Rehabilitation

**DOI:** 10.1007/s11065-021-09531-2

**Published:** 2022-01-25

**Authors:** W.J. Doedens, L. Meteyard

**Affiliations:** School of Psychology and Clinical Language Sciences, University of Reading, Whiteknights, RG6 6AL United Kingdom

**Keywords:** Language use, Functional communication, Aphasia, Multimodal, Interactive, Common ground

## Abstract

Aphasia is an impairment of language caused by acquired brain damage such as stroke or traumatic brain injury, that affects a person’s ability to communicate effectively. The aim of rehabilitation in aphasia is to improve everyday communication, improving an individual’s ability to function in their day-to-day life. For that reason, a thorough understanding of naturalistic communication and its underlying mechanisms is imperative. The field of aphasiology currently lacks an agreed, comprehensive, theoretically founded definition of communication. Instead, multiple disparate interpretations of *functional communication* are used. We argue that this makes it nearly impossible to validly and reliably assess a person’s communicative performance, to target this behaviour through therapy, and to measure improvements post-therapy. In this article we propose a structured, theoretical approach to defining the concept of functional communication. We argue for a view of communication as “situated language use”, borrowed from empirical psycholinguistic studies with non-brain damaged adults. This framework defines language use as: (1) interactive, (2) multimodal, and (3) contextual. Existing research on each component of the framework from non-brain damaged adults and people with aphasia is reviewed. The consequences of adopting this approach to assessment and therapy for aphasia rehabilitation are discussed. The aim of this article is to encourage a more systematic, comprehensive approach to the study and treatment of situated language use in aphasia.

## Introduction

Decades of research in aphasia has predominantly focused on the (impaired) processing of spoken and written words and sentences (Vigliocco et al., 2014). Most of this research has studied language in isolation, in which words and sentences are presented aurally or visually in highly controlled tasks. Notably, measures of language impairment borrowed from studies in psycholinguistics, using decontextualized tasks such as picture naming, repetition, single word, and single sentence comprehension to obtain a linguistically well-defined profile of impairment. Such measures provide a starting point for clinical intervention that aims to repair the parts of language processing that have been impaired (Whitworth et al., 2005). These approaches have formed the foundation for aphasia rehabilitation programs that address the ‘impairment’ present in aphasia, and are effectively and productively used for assessment, therapy planning, and differential diagnoses to this day (Thompson et al., 2008).

In recent decades, psychology, linguistics, and neuroscience have seen a shift away from the assumption that the mind and brain can be understood in isolation through the lens of highly simplified and restrictive tasks (Dhami et al., 2004; Falandays et al., 2018; Hamilton & Huth , 2018; Meteyard & Bose, 2018). This is part of a broader acknowledgement in the cognitive sciences that restricted lab-based ‘in vacuo’ tasks (Clark, 2018b), prized for their high degree of experimental control, may not accurately or validly reflect the cognitive processes that take place in real-world ‘in situ’ situations (Clark, 1996; Clark, 2018a; Hamilton & Huth, 2018; Meteyard & Vigliocco, 2018; Owen et al., 2010; Vigliocco et al., 2014; Willems, 2015). A key indicator of this issue is the fact that performance on restricted, decontextualized tasks (e.g., word production when naming pictures) does not always generalise to situated, more naturalistic tasks that are thought to involve the same cognitive processes (e.g., word production during conversation; Hamilton & Huth, 2018; Herbert et al., 2013; Owen et al., 2010).

In aphasiology, a similar shift from the focus on ‘in vacuo’ to the ‘in situ’ study has taken place in the assessment and treatment of language since the 1970s, when Audrey Holland described the discrepancy between language performance on standardised tests and actual language use for communication in the real world, also referred to as *functional communication*. While it remains central to assess impaired *language* functions, there is an increasing focus on how well someone is able to use language in their everyday lives (Thompson et al., 2008; World Health Organization, 2001). For speech and language therapists, as well as people with aphasia, improvements at the level of functional communication are often the main desired outcome and are central to collaborative goal-setting (e.g., Hersh et al., 2012; Worrall et al., 2010).

There is now general agreement in the aphasia literature that communication^1^ requires a different set of cognitive processes, and a different set of behaviours, when compared to decontextualized ‘in vacuo’ tasks that are traditionally used to measure spoken and written language processing (Beeke et al., 2011; Holland, 1982; see section ‘Language in and out of context’ below). Critically, there is a lack of agreement on how to define functional communication, and therefore how to identify the cognitive processes and behaviours that functional communication requires (Elman & Bernstein-Ellis, 1995; Wallace et al., 2018). This is problematic for the field, as effective assessment and treatment of day-to-day communication is crucial for successful rehabilitation (e.g., Hersh et al., 2012; Worrall et al., 2010). For example, the term ‘functional communication’ has been used inconsistently and in heterogeneous ways across studies (Elman & Bernstein-Ellis, 1995). This has had a negative effect on assessment and intervention practices (Brady et al., 2016; Doedens & Meteyard, 2020; Wallace et al., 2014).

The assessment of both baseline ability and outcomes post-therapy have been affected by inconsistent terminology in the literature. Therapy effects at the level of functional communication are often not measured (Brady et al., 2016; Rohde et al., 2012; Verna et al., 2009), or a set of highly heterogeneous measures are used (Brady et al., 2016; Doedens & Meteyard, 2020; Wallace et al., 2014). This has made it difficult to synthesise, compare, and pool results from different studies. Consequently, it has remained difficult to draw strong conclusions about the effectiveness of therapy at the level of functional communication (Brady et al., 2016; Wallace et al., 2014). Recently, experts have struggled to reach a consensus on a single existing measure for the domain of Communication as part of a core set of outcome for aphasia research (COS-ROMA; Wallace et al., 2018).

The lack of an agreed definition for functional communication also makes planning therapy difficult, both in terms of directly targeting functional communication in therapy, and indirectly targeting functional communication through processes of generalisation^2^. Systematic reviews have shown that speech and language therapy intervention is effective in improving functional communication when compared to no intervention, with moderate effect sizes (Brady et al., 2016). However, consistent, reliable, long-term generalisation effects on functional communication from speech and language therapy are yet to be observed (Brady et al., 2016; Simmons-Mackie et al., 2014; Webster et al., 2015). There is robust evidence that therapy approaches based on ‘in vacuo’ decontextualised language processing skills, such as naming individual pictures, improve performance on words and sentences practiced during therapy (e.g., Brady et al., 2016; Palmer et al., 2019). However, there is limited evidence that improvements observed in therapy generalise to words and sentences that have not been practiced (Efstratiadou et al., 2018; Nickels, 2002; Raymer et al., 2007; Wisenburn & Mahoney, 2009), to tasks that have not been practiced (Boo & Rose, 2010; Conroy et al., 2009; Croot et al., 2014; Herbert et al., 2013), or to different levels of functioning such as discourse (Boyle, 2011; Carragher et al., 2013) or functional communication (Brady et al., 2016; Carragher et al., 2012, 2015). Similarly, it has remained largely unclear as to why some individuals with aphasia show greater therapeutic improvements, and sometimes more generalisation, than others (Best et al., 2013; Lorenz & Ziegler, 2009), and difficult to predict who will respond to what kind of impairment-based therapy (Fillingham et al., 2006; Neumann, 2017; Webster et al., 2015; Wisenburn & Mahoney, 2009). Responsiveness to treatment is mediated by a multitude of components such as dosage and treatment intensity (e.g., Bhogal et al., 2003; Brogan et al., 2020; Doogan et al., 2018), mood, motivation, personal relevance, and the therapist-client relationship (Whitworth et al., 2014), but we believe that a clear understanding of the targeted behaviour is critical for effective intervention.

There is a movement to address the problems regarding assessment, intervention, and generalisation of therapy effects to functional communication (Barnes & Bloch, 2018; Carragher et al., 2012; Harmon, 2020; Hersh et al., 2012; Kagan et al., 2008; Siegert & Taylor, 2004; Webster et al., 2015; Worrall et al., 2011). The aim of this review is to show how an existing theoretical framework of real-world communication can be applied to aphasia rehabilitation, thereby providing the field with a working definition of functional communication. The aim is for the framework to describe a set of general principles that are involved in communication in everyday life, in terms of both internal, cognitive factors and external environmental factors. This approach is nomothetic. We want to make generalisations about functional communication so that we can describe, across different individuals with aphasia, the cognitive and behavioural skills that are relevant to communicative challenges faced in everyday life. Furthermore, we hope such a framework will help disentangle why everyday communication is more difficult for some people with aphasia compared to others (Holland, 1982). To pre-empt the discussion below, we will apply the framework from Clark (1996), who defines situated language use as a face-to-face interaction unfolding in real-time between two or more people, that allows communication via multiple modalities, and is grounded in the context provided by the immediate environment and the relation between the individuals and is built during the course of interaction. We have previously used this framework to evaluate which existing published standardised measure is the most likely to involve functional communication skills (The Scenario Test; van der Meulen et al., 2010; see Doedens & Meteyard, 2020). Table 1 provides a summary of this framework.

**Table 1:** The key components that characterize language in use (based on Clark, 1996)

**Components**	**Definition**	**Sub-components**
*Interactive*	Joint activity between two people. Actions of one person depend on those of the other.	$\bullet$ Feedback or backchannels $\bullet$ Co-construction of dialogue $\bullet$ Common ground
*Multimodal*	Multiple interdependent channels of communication are available and integrate into a single composite message. Different channels replace, supplement, complement, and emphasize speech.	$\bullet$ Language $\bullet$ Prosody $\bullet$ Gesture $\bullet$ Facial expressions $\bullet$ Body posture
*Contextual (relies on common ground)*	Common ground provides interlocutors with context that allows them to assume a degree of “givenness” of information, or directly use physical referents during communication. This relieves the communicative burden.	Pre-existing: $\bullet$ Communal common ground $\bullet$ Personal common ground Discourse representation: $\bullet$ Situational context $\bullet$ Communicative context

## Language In and Out of Context

The gap between decontextualised ‘in vacuo’ language tasks and functional communication has been critiqued within aphasiology for a long time (Armstrong, 2009; Barnes & Bloch, 2018; Hersh et al., 2012; Holland, 1977; Kagan et al., 2008; Worrall et al., 2011). Decontextualised tasks are typically at the level of single words and single sentences, with a focus on response accuracy. They are necessarily a simplified version of language production or comprehension. For example, a person may be asked to provide single word names for a series of unrelated pictures, to repeat a list of single words or single nonwords, to match a spoken word or sentence to presented pictures, or to produce a list of words that belong to a given category (Goodglass et al., 2001; Kertesz, 2009). Such tasks can be extended to measures of discourse, as in complex picture description, procedural narratives, or paragraph comprehension.

We can delineate the ways in which these tasks differ from typical everyday communication. Perhaps the most obvious difference is that to move from an in-vacuo task to a situated task we have to add things, and this necessarily makes a situated task more complex than an in-vacuo task. In reality, there is a continuum from isolated, decontextualised language tasks to contextual, situated language tasks, but for illustration we will compare the difference between these types of tasks using the analogy of someone ordering a drink at a café. Anecdotally, this is an activity that speech and language therapists often will use as part of a functional intervention with a person with aphasia. First, in decontextualised tasks, beyond the interaction with the therapist or person who is administering the task, there is usually minimal interaction that is measured or is part of the language task itself (Barnes & Bloch, 2018). This is best exemplified by the fact that many or all of established measures of language impairment used in aphasiology can be administered using an automated computer programme (Palmer et al., 2019; Westbury, 2015). If the same were true for ordering a drink, the accurate comparison would be someone using a vending machine. Second, the goal of the task is circumscribed and is typically a process of verification, for example producing the right picture name, or correctly choosing a matching picture. In studies of everyday communication, it has been argued that language has both a transactional and interactional purpose (Brown & Yule, 1983; Simmons-Mackie & Damico, 1995; Simmons-Mackie, 1998). That is, whilst we seek to exchange (usually) accurate information using language, we also build and maintain relationships, and achieve social, emotional, and participatory goals when we communicate (Armstrong, 2009; Barnes & Bloch, 2018). It is important to note that many people, including ourselves, view the transactional and interactional functions of language as simultaneous and inseparable (Armstrong & Ferguson, 2010; Ferguson & Thomson, 2008; Halliday, 2004). The person in the café will order their drink by offering linguistic information, such as the name of the drink they want, but they may also want to interact appropriately with the barista, make some polite conversation, and say “thank you”. Third, there is usually no substantive context that precedes an ‘in vacuo’ task or that builds up during the task. In the café, the person approaches and enters the café environment, can search for the menu and the items on offer, and rely on their previous experiences in similar places. In an ‘in vacuo’ task, each subtest in an impairment-based assessment can stand alone and is unrelated to the tasks that come before or afterwards. Similarly, the items in a test such as the words, sentences or pictures, will usually be unrelated to items that come before or after. Often, this is done deliberately, through randomisation and counterbalancing to prevent the influence of one item on the next. Finally, decontextualised tasks are often limited to one channel of information transfer, input or output (e.g., verbal, written, or gesture). This is deliberate and necessary to isolate some aspect of processing in language production or comprehension. In real life, a person can rely on different ways of expressing themselves. In a café, a person can ask for a coffee, point to the previous customer or menu to indicate wanting a similar order, respond with a nod or brief ‘yes’ to a list of options offered by the barista, or do a combination of these things.

The data gained from decontextualised tasks is “thought to be representative of the language skills of the individual that are then ‘used’ in everyday discourse” (Armstrong, 2009; p.143.), and correlations between different measures support this inference. Individuals with non-fluent aphasia show difficulties producing verbs when naming pictures and during conversation (e.g., Carragher et al., 2013). Accuracy on a picture naming task has been shown to positively correlate with the number of nouns and content words produced during a conversation (Herbert et al., 2008), although this correlation was not observed in a similar study by Mayer and Murray (2003). In a large sample of people with aphasia (n=67) scores on a standardised set of decontextualised tasks (the Western Aphasia Battery; Kertesz, 2009) correlated positively with communication as measured by the CETI (Communication Effectiveness Index; Bakheit et al., 2005). So, we can say with some certainty that these ‘in vacuo’ tasks reflect the performance of people with aphasia during everyday communication, for example, when considering the amount of language they might produce. Decontextualised, in vacuo tasks do provide critical pieces of information that allow a particular aphasia profile to be categorised, such as agrammatism or jargon aphasia (Hillis, 2007), and hypotheses to be generated about the cause of the language difficulties experienced by a person with aphasia (e.g., Robson et al., 2012; Thompson et al., 2003).

However, taking data from a collection of decontextualised tasks is not sufficient to understand how someone communicates day-to-day (Holland, 1980). Decontextualised tasks do not provide an understanding of the *demands* of everyday communication or *the skills needed* to communicate effectively in the real world. As noted above, situated, functional communication tasks have additional elements that we need to understand. Some of these will benefit language processing (e.g., increased context; Skipper, 2014), some may make language processing more challenging (e.g., spontaneous, time limited interaction with another person; Barnes and Bloch, 2018), and others may be a benefit or a cost depending on the circumstances and the individual (e.g., multimodality, Vigliocco et al., 2014). Everyday communication requires different parts of language processing to work in concert, and to work alongside other cognitive skills such as attention, working memory, and executive control processes (e.g., Carragher et al., 2012; Salis et al., 2017).

The continuum from in vacuo tasks to in situ tasks can be seen in the literature on generalisation of lexical retrieval (Conroy et al., 2018; Webster et al., 2015), functional rehabilitation for people with aphasia (Byng et al., 2013), and in the protocols of published trials of aphasia rehabilitation (Breitenstein et al., 2017). For example, a hierarchy begins with a ‘simple’ task that is in vacuo (e.g., repetition and reading aloud), moving to production of the same items with less support (still in vacuo, for example, production from a picture cue with no repetition or reading aloud), then introducing more complexity that begins to situate the task (e.g., production in response to a question, which introduces some context and an interactive element), and finally a demanding task that resembles day-to-day communication (e.g., production during a role play; e.g., Bilda, 2010; Breitenstein et al., 2017; Meteyard et al., 2014). In order to fully understand how to *directly target* functional communication in therapy, or to hypothesize how therapy might generalise to the level of everyday communication, a thorough understanding of functional communication is needed. That is, what cognitive and linguistic processes do we need to target and how should we target them? In response to this question, there are increasing data from therapeutic studies that show that functional communication needs to be targeted *directly*, and that generalisation from in vacuo decontextualised therapy tasks to functional communication cannot be relied upon (e.g., Goral & Kempler, 2009; Kempler & Goral, 2011; Stahl et al., 2016).

A principal goal of applying a framework to functional communication is to understand how specific linguistic and cognitive impairment profiles respond to the challenges of everyday communication, that is, why everyday communication is more difficult for some people with aphasia than for others. Such knowledge would enable researchers and clinicians to identify specific cognitive and cognitive-linguistic skills as potential targets for assessment and rehabilitation, just as under the impairment-based approach, phonological and semantic processes are key drivers for therapy (Meteyard & Bose, 2018).

## Holistic Views of Aphasia

A major and positive shift in aphasia rehabilitation occurred with the application of the WHO International Classification of Functioning (ICF, World Health Organization, 2001, e.g., Simmons-Mackie & Kagan, 2007; Worrall et al., 2011). The ICF brings together medical and social models of disability to provide a holistic account of the impact a particular condition has on an individual. The degree to which an individual is able to function arises from the interaction between their health condition, such as a disease, disorder or injury, and personal and environmental factors, such as individual coping styles or the physical environment. Functioning is defined according to different components that are classified as medical or physical, social and emotional. Anatomical, physiological, and psychological functions are represented in the ‘Body Functions and Structures’ component. This reflects “problems in body function or structure such as significant deviation or loss” (p.10; World Health Organization, 2002), and rehabilitation aimed at the impairment attempts to “prevent or ameliorate limitations in person or societal functioning by correcting or modifying intrinsic functions or structures of the body” (p.8, World Health Organization, 2002). Impairment-based language processing skills, such as reception of spoken language or expression of written language, fall into the ‘Body Function’ component of the ICF. Historically, interventions targeted at the ‘impairment’ have been seen as the most desired – if the underlying issues with language processing can be addressed, the aphasia itself should become less severe. We can consider ‘impairment-based’ interventions to be those that address the neurological damage, for example, reperfusion of the cortex to reduce the extent and severity of the lesion (Hillis, 2007). In aphasia rehabilitation, the impairment is also strongly associated with cognitive-neuropsychological models of language processing (Galletta & Barrett, 2014; Thompson et al., 2008). For example difficulties with word retrieval may be caused by problems accessing phonological or semantic information (Galletta & Barrett, 2014; Meteyard & Bose, 2018).

The social and emotional function components of the ICF include ‘Activities’ (such as the ability to complete a task), and ‘Participation’ (such as inclusion or involvement in a given life event or situation, often with a focus on social or societal participation). Part of the difficulty of working with functional communication is that it extends across the Activities and Participation components of the ICF (WHO, 2001; ‘d3 communication’ in the ICF 2001, e.g., Kagan et al., 2008; Wallace et al., 2017). The difference between Activities and Participation components can be seen as existing on a continuum. For example, ‘asking a question’ and ‘expressing one’s feelings’ can be seen as specific communicative activities (Simmons-Mackie & Kagan, 2007). Similarly, ‘having a conversation’ might be categorised as a specific activity, while ‘socialising’ or ‘engaging in social life via communication’ might be described as fitting in the ‘Participation’ component. All these examples are part of what is ‘functional communication’.

In recognition of the far-reaching impact of communication difficulties, evidence-based holistic approaches to aphasia rehabilitation are becoming the gold standard. These approaches stress the importance of personally relevant and functional goals (Hersh et al., 2012; Kagan et al., 2008; Siegert & Taylor, 2004; Worrall et al., 2011), seek to maximise quality of life outcomes (Simmons-Mackie & Kagan, 2007), and emphasise a comprehensive approach to rehabilitation that targets all components of the ICF (Rose et al., 2013a). To target functional communication effectively through therapy, it is critical for the field to agree upon a definition of what functional communication is.

## Existing Frameworks of Functional, Real-World Communication

It is worth briefly reviewing available frameworks of functional communication in order to justify the choice of using the framework by Clark (1996). A wide range of disciplines have developed models of functional communication, including psychology, linguistics, communication science, sociology, neuroscience, and engineering. This reflects the multifaceted nature of communication. The now classic Shannon-Weaver model (1949) aimed to describe the transmission of a message and the influence of potential distortions such as ’noise’ (also described as the level of uncertainty) on that message. Although very successful in optimising the process of information transmission, the model has been criticised for describing communication as a linear process, solely running from speaker to listener, rather than an interactive process between a speaker and a listener, for omitting contextual environmental factors, and for ignoring the relational social aspects of communication between two people (Kincaid, 1979). Following this, a great number of models were proposed that often expanded the original version. For example, Schramm (1954) added an encoding and decoding element to the model to capture the way meaning is transmitted and interpreted between two people. Westley and MacLean (1957) added feedback and multiple channels for communicating, and highlighted the influence of the wider environment to communication. Berlo’s Sender-Message-Channel-Receiver model (SMCR, 1960) included contextual factors that influence communication, such as communication skills, attitudes, and social support. Overall, these models remained relatively linear in design, still describing communication as a speaker to listener ‘left-to-right’ process. Although not quite interactive, the Ruesch and Bateson model (1951) described the different levels of complexity through which human communication can take place, ranging from an intrapersonal level to a cultural level. From a more philosophical perspective, Alexander (1988) describes similar influences of experience and attitude of the speaker and listener on the interpretation of the message. This model also describes the potential misunderstandings that can result from differences in these experiences and attitudes.

In one of the first models applied to aphasia, Wepman et al. (1960) described where different neurological language and speech disorders originate, based on data from people with aphasia. Three basic processes were presented, namely, the input of signals to the ears, eyes, and body to the brain, the integration of these signals into a cognitive symbolic formulation, and the output of a signal through the ears, eyes, hands, and body. As it stands, this model describes the process of communication at the level of an individual, excluding the interactive and environmental aspects of communication.

In the 1950s, language processing models emerged in psycholinguistics. These models focused on how words and sentences are produced and understood, typically excluding multimodal or interactive aspects of communication (Vigliocco et al., 2014). Highly controlled lab-based experiments were used to understand the components of language processing in great detail. From this research, influential models such as those by Levelt (1989), Patterson and Shewell (1987), Dell (1986), and Ellis and Young (1988) were published. By-and-large, these models focused on the production and comprehension of single words, with some also describing sentence-level processing (Caramazza & Zurif, 1976; Schwartz et al., 1980). These word- and sentence processing models gave rise to decades of fruitful language research. In addition, these models have heavily influenced the nature of language and communication research in general, and research on aphasia more specifically (e.g., Kay et al., 1996). Alongside the emergence of single-word processing models, researchers such as Kendon (1980), McNeill (1992), and Goodwin (1995) emphasised the multimodal nature of information exchange, in which non-verbal channels such as gesture, facial expression, and body movement complement and supplement verbal language. Gesture has since become a fruitful area of research in aphasia (e.g., de Beer et al., 2017, 2020; Roper et al., 2016; van Nispen et al., 2017; see section *Language use is multimodal* below).

Different branches of linguistics have provided alternative understandings of how individuals use language to communicate. Systemic Functional Linguistics (SFL; 2004) is a sociolinguistic approach that sees language as a system that enables people to create meaning. Individuals make choices each time they communicate, and the semantic and syntactic choices available to a person are influenced by context. Context, in this framework, is defined as extralinguistic aspects such as field (the nature and content of the situation a person finds themselves in), tenor (the interlocutors involved in the exchange, their relationship, etc.), and mode (the way in which language is used, such in writing, speaking, on the phone, etc.; Armstrong, 2005). SFL describes the ways in which communicative settings might differ in everyday life, such as across different groups and conversation topics. The focus of analysis in SFL is usually on language production rather than comprehension, in an attempt to understand how contextual differences influence specific aspects of production, such as word choice, sentence structure, and coherence. This approach can incorporate the multimodal aspects of discourse, such as the use of images or the physical context (Baldry & Thibault, 2017). SFL focuses on language as a whole system, so the intention is not to describe individual cognitive or linguistic processes. SFL has been applied to the study of aphasia (e.g., Armstrong, 2000; Ferguson, 1992) and used to annotate and describe how individuals with aphasia use language (Ferguson & Thomson, 2008). A sociological framework of communication has also been applied to the study of everyday communication in aphasia (e.g., Beeke et al., 2007, 2011; Wilkinson, 1995). The focus of conversation analysis is to study face-to-face conversation, to understand how social interaction takes place and how the interaction is organised through conversation, for example, how repair takes place when there is a breakdown in the conversation. The conversation analysis methodology uses spontaneous, interactive, natural conversation as its object of study (Beeke et al., 2007), with careful annotation of natural samples of conversation (Damico et al., 1999; Sacks et al., 1974). Multimodal communication can be incorporated as part of the annotation and analysis process, but the semiotic and linguistic origins of conversation analysis means that this method is not concerned with how meaning might be processed ‘inside’ the mind (i.e., cognitively; Enfield, 2013). In aphasia rehabilitation, conversation analysis has been used to develop interventions targeted at analysing and improving the interaction between people with aphasia and their conversation partner (i.e., ‘conversation partner training’, e.g., Barnes & Nickels, 2017; and see Section *Language use is interactive* below). Finally, within the aphasia literature, conversation analysis is often applied as an ideographic approach. Rather than seeking to generalise across a group of people with aphasia, this method focuses on how talk is organised in a dyad, stressing the importance of the individual differences that are present, such as the difference in impairment profiles (Barnes & Bloch, 2018).

In summary, each of the aforementioned models and frameworks aim to understand part of the communication process. Each does so by studying one aspect of communication in detail, such as word processing, gesture, language use, or talk-as-interaction. Our interest is in understanding the cognitive and cognitive-linguistic demands of everyday communication, so that the same analytic rigour that we see in the impairment-based approach through an analysis of cognitive mechanisms and processes can be applied to functional communication. As such, we needed a comprehensive definition of communication that will help us to understand its defining qualities.

## A Definition of Situated Language Use

In this section of the paper, the framework of *situated language use* according to Clark (1996) will be described. Clark (1996) defines the general principles as (1) interactivity, (2) multimodality, and (3) embeddedness in a physical and communicative context, as well as a context of common ground. These three factors will be described in more depth below. In addition to providing a definition of everyday communication, Clark (1996) also provides a structured approach to investigating the different forms that communication can take on in day-to-day life, such as speaking with one compared to more individuals and writing a letter compared to speaking to someone in person. By defining a *basic setting of situated language use*, Clark (1996) opens up avenues for experimental, systematic investigations of everyday communication, and provides a framework by which to understand the relationship between different forms of communication that are encountered in day-to-day life.

### The Basic Setting of Situated Language Use

Communication in everyday life varies across settings, modalities, and ways of communicating, from speaking with a sibling at home, listening to an audio book in the car, performing for an audience in the theatre, writing a letter to a friend, or ordering a meal in a restaurant. The ability and manner in which one communicates across these different settings will vary depending on a number of factors, such as the circumstances or personal motivation (Harmon, 2020; Ramsberger & Menn, 2003). To evaluate the principles that govern situated language use, researchers have started by studying this phenomenon in its most basic setting, namely, face-to-face communication (Barnes & Bloch, 2018; Bavelas & Chovil, 2000; Clark, 1996; McDermott & Tylbor, 1983; Pickering & Garrod, 2004). Face-to-face communication is the most commonly used and pervasive form of communication. It is universal to all human societies, it is the basis for typical language acquisition in children, and it does not require education or special skills (Bavelas & Chovil, 2000; Clark, 1996). Indeed, Davidson et al. (2008) showed that face-to-face conversation is the most frequently occurring communicative activity in daily life for people with aphasia. It therefore makes sense to begin by studying communicative skills in this setting (Kagan & Simmons-Mackie, 2007; Ramsberger & Menn, 2003). The reasoning is that once the principles that govern face-to-face communication are teased out, and a person’s communicative skills in this basic setting are assessed, language use in other communicative situations can be derived from the basic face-to-face exchange based on a set of pre-determined parameters (Clark, 1996). Examples of such parameters are the immediacy of time (e.g., face-to-face communication takes place in real-time, while a video chat may occur with delays) and physical presence (e.g., during face-to-face communication participants see each other without obstruction, while speaking on the phone excludes visual information. For a more detailed discussion of these parameters see Clark, 1996).

### Language Use is Interactive

Communication is a joint activity (Barnes & Bloch, 2018; Clark, 1996; Schegloff, 1982), meaning that language is achieved by two or more people who coordinate their actions to achieve a common goal. Every decision made during a conversation will depend on the actions of the other. Language use is therefore an inherently interactive process, in which two or more participants work together and coordinate their actions to create meaning. The whole, as well as the actions of each individual, can be studied within that process. When language production and comprehension are studied outside of the interactive process, for example, in isolation or based on the behaviour of a single person, this may involve inherently different cognitive processes and behaviours as compared to language when it is used for communication. The presence of another interlocutor brings a set of factors that influence the overall process such as the shared knowledge between interlocutors. This will be discussed in the section on *personal common ground* below.

### Language Use is Multimodal

Communication is a fundamentally multimodal phenomenon (Bavelas & Chovil, 2000; Clark, 1996; Kendon, 1980; McNeill, 1992; Vigliocco et al., 2014). This means that information is communicated through the vocal modality (e.g., speech) as well as the visual modality (e.g., gesture). Within each modality, different channels of expression are used for communication, such as the lexico-syntactic and prosodic channels in the vocal modality, and facial expressions, gesture, and body movements in the visual modality (Stivers & Sidnell, 2005; Vigliocco et al., 2014). These modalities and channels interact and are interdependent, becoming a single composite message. By studying language (i.e., the lexico-syntactic channel) in isolation, the complexity and interdependence of the different channels is overlooked (Vigliocco et al., 2014), and a wealth of information that is relevant for communication is missed.

### Language Use is Based on Common Ground

Finally, communication in a real-world setting allows interlocutors to rely on context (Clark, 1996). Defining context is a difficult task, because the concept is broad and all-encompassing (Meteyard & Vigliocco, 2018). Clark (1996) refers to context for situated language use as common ground, being the set of shared knowledge, beliefs, and assumptions that exists between two speakers. Everything that is understood by two interlocutors engaged in conversation is part of their common ground (Clark, 1996). When two interlocutors do not understand each other, conversation breaks down, or is shifted to a different topic. For example, if one speaker says “my dogs”, both interlocutors must ensure they share the belief that this refers to the speaker’s feet, rather than his pets, for that particular conversation to be successful (Clark & Brennan, 1991). If the listener has not understood, both parties must work together to ensure sufficient mutual understanding is achieved to continue the conversation. Common ground can be established before two people start interacting (“pre-existing common ground”), and it can develop while two people interact (“discourse representation”). For example, the reference *small candle* can be understood as referring to a particular candle if (1) of the candles physically present, one is smaller than the others (“situational context”), (2) a candle was previously spoken about during the same conversation (“communicative context“), or (3) the interlocutors have a shared personal experience or are members of a community in which the particular small candle is well-known (“personal or communal common ground”; Kronmüller & Barr, 2015).

1. Communal common ground: Communal common ground refers to shared beliefs and knowledge based on a shared nationality or religion. Customs that are specific to a certain country or culture will be shared and readily understood between people from that culture.
2. Personal common ground: Personal common ground reflects the number of shared experiences two participants have had together, also referred to as the level of acquaintedness or familiarity.

Discourse representation

1. Situational context: The situational context includes what is physically present in the perceptual environment.
2. Communicative context: The communicative context is an accumulation of what has been referred to earlier in conversation (through any modality or channel of expression).

## A Review of the Literature on Situated Language Use

Clearly, language use in everyday life is a complex, multi-faceted, dynamic phenomenon. The framework provided by Clark (1996) provides us with parameters to guide our discussion of this topic (as shown in Table 1). The framework is not new, nor are many of the ideas included in the framework. The novelty lies in applying this framework to aphasia rehabilitation. Over the past decades, much research has been done on communication with healthy adults in the fields of communication science, psychology, linguistics, neuroscience, psycholinguistics, and sociology. To get a better understanding of the complexity and variety of processes that occur during communication, we will review the literature on the effect of each component on communication. In order to get a sense of the amount of work that has been completed, as well as where the gaps in knowledge are, we will summarise what we know from research with healthy adults as well as with people with aphasia separately.

Due to the multitude of different topics covered in this theoretical narrative review, a more restricted systematic approach was not possible. Instead, guided by Clark (1996), the authors searched databases for highly cited papers, as well as the most recent existing review articles on each topic. Based on the database searches and reference lists of the papers found, a collection of important findings relevant for the current discussion are summarised below.

### Language Use is Interactive

#### Neurologically Healthy Controls

Research with neurologically healthy controls shows that a person’s communicative efficiency when giving instructions is better on an interactive task (i.e., with another interlocutor present who listens and provides immediate feedback) compared to a non-interactive setting (i.e., without another interlocutor present; Clark & Krych, 2004). This is due to the availability of feedback and the co-ordination of actions between two interlocutors. Studies have shown that interlocutors help each other in creating meaning and dialogue by providing each other with feedback (Brunner, 1979; Clark & Brennan, 1991; Clark & Krych, 2004; Schegloff, 1982). Listeners can provide immediate feedback during communication by providing so-called ‘backchannels’, also referred to as ‘minimal turns’ or ‘continuers’. These include signals such as “uh huh”, “right”, “okay”, nods, smiles, or frowns. Backchannels are expressed through any channel or modality, as discussed in the next section, to indicate attentiveness and involvement, comprehensibility of the message, and the listener’s emotional response to the content (Brunner, 1979; Clark & Brennan, 1991; Clark & Krych, 2004 Schegloff, 1982). Speakers have been shown to monitor listeners for backchannels, and adjust their messages depending on the type of feedback they observe, for example, the need to rephrase or elaborate on the message (Clark & Krych, 2004; Tolins & Fox Tree, 2014). Backchannels have been shown to influence the content of speaker dialogues. Different types of backchannels can lead to significantly different ways in which a story is told, while the absence of certain backchannels can result in less climactic endings to stories, qualitatively worse story content, and modulation of abstract language use (Bavelas et al., 2000; Beukeboom, 2009; Norrick, 2010; Tolins & Fox Tree, 2014).

While the presence of feedback in an interactive exchange is helpful, it also presents interlocutors with the challenge of monitoring one’s conversation partner for such feedback *while* communicating a message. This dual-task of simultaneously managing production processes and monitoring the other conversation partner, is likely to make interactive communication a more cognitively demanding task compared to non-interactive communication.

Similarly, an important consequence of interactive communication compared to non-interactive communication is that comprehension and production processes intersect. That is, while one interlocutor expresses something, the other listens and simultaneously plans their response (Clark, 1996; Levinson, 2016; Levinson & Torreira, 2015). The simultaneous occurrence of language production (e.g., planning for word and sentence production, self-monitoring) and language comprehension processes (e.g., semantic and syntactic processing), that are each cognitively demanding themselves (e.g., Roelofs, 2011), makes language processing in an interactive setting more cognitively demanding than language processing in non-interactive settings (Levinson, 2016).

The presence of a conversation partner also brings the pressure of time (Barnes & Bloch, 2018; Carragher et al., 2012; Clark, 1996). Interactive communication is coordinated by, among other things, transitions between who speaks and who listens. This turn-taking between interlocutors is governed by specific time-restrictions that require each individual to respond within what is considered a ‘reasonable’ time frame, leaving little time for extensive planning of a response (Clark, 1996; Conroy et al., 2018; Levinson, 2016; Sacks et al., 1974). As a result, the interactive component of situated language use poses cognitive challenges for the interlocutors that are not present in a non-interactive setting.

Finally, the presence of another person brings new dynamics and variables into the communication process, compared to when an individual attempts to express something without another person present (e.g., as during monologue tasks). The influence of knowledge, beliefs, and experiences that are shared between interlocutors will be discussed in “Communal and Personal Common Ground”.

#### People With Aphasia

Most of the research on interactive aspects of communication in aphasia have focused on (1) the shared responsibility in communicating when conversation breaks down, and (2) the influence of the communication partner on the communicative competence of the person with aphasia.

The shared responsibility of constructing conversation between interlocutors has received much attention in the aphasia literature (Goodwin, 1981, 1995; Milroy & Perkins, 1992; Simmons-Mackie et al., 2014). A large body of research has focused on conversational repair, namely, how problems or breakdowns in conversation are dealt with by the person with aphasia and their conversation partner (Lindsay & Wilkinson, 1999; Milroy & Perkins, 1992; Schegloff, 1982). Often, this is done by analysing turn-taking patterns during conversation, which, according to conversation analysis principles, can reveal how people understand and respond to each other during interaction (Beeke et al., 2007; Sacks et al., 1974; Schegloff, 1982). Through this approach, research has shown that repairs in conversation are often different for people with aphasia compared to neurologically healthy controls. Conversational repairs can take longer for people with aphasia and their conversation partners, and more often rely on collaborative repair rather than on the efforts of a single interlocutor (Beeke, 2012; Lubinski et al., 1980; Milroy & Perkins, 1992). As such, the interactive component of face-to-face communication means that people with aphasia can rely on the conversation partner in the co-construction of dialogue and meaning in communication when experiencing problems in conversation (Beeke et al., 2007; Booth & Perkins, 1999; Lindsay & Wilkinson, 1999; Oelschlaeger & Damico, 1998). For example, research has shown that conversation partners may help people with aphasia in completing conversational turns when word finding difficulties occur (Bloch & Beeke, 2008; Oelschlaeger & Damico, 1998), and in repairing turns when the person with aphasia experiences a communication breakdown more generally (Lindsay & Wilkinson, 1999; Perkins, 1995; Samuelsson & Hyde, 2016). This means that for some people with aphasia, due to the presence of communicative difficulties, more of the efforts to achieve successful communication, or the conversational burden, lie with their conversation partner compared to neurologically healthy controls (Linebaugh et al., 2006). Indeed, Linebaugh et al. (1982) showed that greater communicative impairment (as measured on the Communication Activities of Daily Living, Holland, 1980) was associated with a greater shift of conversational burden.

Numerous studies have focused on how a conversation partner can facilitate or inhibit communication for people with aphasia. The response to breakdowns in conversation vary depending on the conversation partner, as people with aphasia show different patterns of conversational repair depending on who they are conversing with. This difference has been shown across types of conversation partners, such as speech and language therapists and people with aphasia’s spouses (Laakso, 2014b; Lindsay & Wilkinson, 1999; Perkins, 1995). The difference between these two groups of conversation partners has often been explained by the ‘institutional’ nature of conversation with the speech and language therapist, while conversation with a spouse is more peer-like in nature. Some research suggests that individual characteristics of the conversation partner, such as their executive function skills, influence their ability to provide communicative support for the person with aphasia (Eriksson et al., 2016). In addition, differences in the amount of shared knowledge and individual discourse styles have also been proposed as possible explanatory factors (Ferguson, 1994; 1998; Green, 1982; Howe et al., 2008; Laakso & Godt, 2016; Perkins, 1995; Wirz et al., 1990). Research on the influence of speaker familiarity on communication will be discussed in “Communal and Personal Common Ground”.

A larger body of research has shown that expanding the conversation partner’s knowledge of aphasia and training them to use communication strategies can improve overall communication with people with aphasia (e.g., Cruice et al., 2018; Howe et al., 2008; Kagan et al., 2001; Lesser & Algar, 1995; Lindsay & Wilkinson, 1999; Nykanen et al., 2013; Pound et al., 2000; Rayner & Marshall, 2003; Simmons-Mackie et al., 2010; Wilkinson & Wielaert, 2012). Most of these studies are based on the idea that communication strategies employed by the non-aphasic conversation partner can create an environment that enables the person with aphasia to communicate optimally. Put differently, changes in the conversation partner’s behaviour can reveal communicative competence in the person with aphasia (Kagan et al., 2001; Turner & Whitworth, 2006). Indeed, these studies show changes in the person with aphasia’s degree of participation in conversation (Kagan et al., 2001; Wilkinson et al., 2010). Based on these findings, a number of therapies have been developed with the intention of improving the conversation partner’s skills to facilitate conversation and to reveal the person with aphasia’s communicative competence (Kagan et al., 2001; Simmons-Mackie et al., 2010). A slightly different approach has emphasized the collaborative aspect of conversation and the importance of training both the conversation partner and the person with aphasia to use communicative strategies (e.g., Beckley et al., 2017; Lock et al., 2001; Nykanen et al., 2013; Simmons-Mackie et al., 2010; Wilkinson et al., 2010; Wilkinson & Wielaert, 2012). These studies have provided support for the idea that therapy can be used to directly influence communication between the person with aphasia and their conversation partners.

Much of the research above focuses on the role of the conversation partner during conversation. Less research has explored the communicative skills of the person with aphasia in an interactive setting, such as self- and other monitoring, the use of feedback, backchannels, and the ability to effectively use compensatory strategies in communication. The impact of feedback provided by the conversation partner, as discussed for neurologically healthy controls, is a helpful element of conversation because it provides an interlocutor direct insight into the comprehensibility of their message. On the other hand, monitoring for feedback can be viewed as an additional cognitive task that might make communication more cognitively demanding.

A few studies have investigated the types of explicit feedback provided by speech and language therapists during intervention, such as direct or delayed comments on the effective use of communicative strategies like drawing or writing (Beckley et al., 2017; Horton, 2008; Simmons-Mackie et al., 1999). This is often unnatural therapeutic feedback, which is not relevant for the discussion of spontaneous communication in everyday life. A number of smaller experimental studies investigated the ability of people with aphasia to respond to expressions of misunderstanding from communication partners during conversation. The findings suggest that, depending on the severity of the aphasia, people with aphasia were generally able to respond to these forms of feedback adequately (Busch et al., 1988; Kovar & van Denmark, 1983; Newhoff et al., 1982). In a large study on communication skills in conversation in patients with left and right hemisphere strokes where the presence of aphasia was not specified, Rousseaux et al. (2010) report a relative preservation of the ability of people with a left hemisphere stroke to attend to their interlocutor for engagement in the conversation and to manage nonverbal feedback from their conversation partner. Whether the ability of people with aphasia to respond effectively to feedback from the conversation partner is mediated by cognitive impairments remains relatively unknown. In the same study, producing feedback was also found to be preserved, which suggests that these patients “were still able to use it to partially encompass their difficulties in understanding the interlocutor” (Rousseaux et al., 2010, p. 1105). Perkins (1995) reported on three people with aphasia who used *minimal turns* such as ‘mm hm’ effectively to contribute to the conversation without using elaborate turns that require the use of more complex linguistic resources. Furthermore, Walker et al. (2016) reported on the production of backchannels by people with aphasia to display different levels of understanding in conversations with their speech and language therapist, such as the production of ‘oh’ at the beginning of an utterance. Subtle differences were found between backchannels produced by the person with aphasia that indicated *claims* of understanding, and the more definitive *displays* of understanding. Speech and language therapists were shown to be sensitive to these differences, responding with elaborations to *claims*, while affirming or changing of topic in response to more definitive *displays* of understanding (Walker et al., 2016). The feedback provided by the person with aphasia thus influenced the course of conversation and helped to ensure mutual understanding. These studies suggest that the use of minimal turns can be preserved in people with aphasia, and can be used by people with aphasia to maintain a natural pattern of turn exchanges during conversation, despite the difficulty in producing linguistic content (Simmons-Mackie & Damico, 1997). Self-correcting one’s errors during conversation, also described as self-repairs, can be seen as an overt form of monitoring during conversation. Repairs in conversation have been studied as a way of understanding where ‘troubles’ in conversation for people with aphasia come from, and how these are resolved by the interlocutors, as discussed above (Beeke, 2012). These studies have shown that, similarly to neurologically healthy controls, people with aphasia show a preference for correcting their own mistakes during spontaneous conversation. The preference for ‘self-initiated, self-repairs’, suggests that on the surface self-monitoring may be largely intact for some people with aphasia (Beeke, 2012; Wilkinson et al., 2003). Overall, there is evidence that people with aphasia are able to monitor conversation, and produce and comprehend interlocutor feedback and backchannels. However, the evidence base remains relatively limited.

One intervention study showed that the use of feedback such as backchannels can be explicitly trained during conversation therapy for dyads. Beeke et al. (2011) report on a therapy in which a person with aphasia was successfully trained to signal verbally that he was still actively working on his turn, for example by saying “um” or “erm”, and non-verbally, such as through grimacing or raising of the eyebrows. Usually, the person with aphasia would leave long pauses during which it was unclear whether he intended to continue his turn or not. Findings such as these underline the important role of feedback in communication, and show that the use and understanding of feedback during communication directly or indirectly can be affected by the presence of aphasia. Furthermore, the provision of feedback can, to some degree, be trained through therapy. More research is needed, however, to explore whether people with aphasia use and can benefit from these interactive components of communication, in both production and comprehension.

Time pressures that are naturally present in typical face-to-face communication make this process more complex and dynamic compared to language processing in isolated, decontextualized tasks such as word-reading and picture-naming (Carragher et al., 2012; Conroy et al., 2018). On various decontextualised language processing tasks, people with aphasia have shown delayed response times compared to neurologically healthy controls (e.g., Crerar, 2004, in word retrieval Galletta & Goral, 2018; Faroqi-Shah & Gehman, 2021 and lexical access and syntactic processing Love et al., 2008). Preliminary studies suggest that delays in processing speed observed in aphasia can lead to problems in the simultaneous activation of information from different domains (e.g., syntactic or lexical), required for comprehension of longer segments of speech (e.g., Conroy et al., 2018; DeDe & Salis, 2020; also described in resource-based accounts such as Avrutin, 2000; Kolk, 2006; Miyake et al., 1994). It is likely that delays in processing speed during language production and comprehension will negatively affect face-to-face communication, given the inherent time pressure in face-to-face interactions. Finally, the presence of a conversation partner has been suggested to influence the communication style, and potentially the communicative effectiveness, of people with aphasia (Simmons-Mackie and Damico , 1995; Simmons-Mackie, 2018). Communication Accommodation Theory (e.g., Gallois & Giles, 2015), in line with theories of *audience design* (Bell, 1984; Clark & Murphy, 1982), posits that interlocutors adjust their talk and communication style to adapt to their conversation partner. This adjustment can occur in speech, pronunciation, nonverbal communication such as gesture or body posture, timing, and discourse style, for example. Simmons-Mackie (2018) suggests that this process can negatively affect communication effectiveness for people with aphasia. People with aphasia may refrain from using unusual yet helpful communication strategies such as gesture, drawing, or writing in an attempt to adapt their communication style to that of their conversation partner. If their conversation partner does not have aphasia, their communication style will more often than not rely heavily on verbal communication.

### Language Use is Multimodal

In clinical and academic settings, it is generally accepted that people with aphasia use other channels of information such as gesture, facial expressions, body posture, body movement, and prosody, in addition to the impaired lexico-syntactic channel, to achieve successful communication (Geigenberger & Ziegler, 2001; Goodwin, 1995; Laakso, 2014a; Pound et al., 2000; Rose et al., 2013b; van Nispen et al., 2017). Interestingly, however, a large number of studies that attempt to capture functional communication do not systematically consider *all* these channels for communication. Very few studies have considered the interplay of all channels that are thought to be involved in communication. Instead, separate fields of research have evolved, each focusing on the use of a specific channel for communication, such as gesture or facial expressions. A brief overview of the relevant research on each channel and its function in communication in neurologically healthy controls and people with aphasia will be presented.

#### Gesture

##### Neurologically Healthy Controls

The field of gesture in neurologically healthy controls is abundant, and a thorough review of the literature is beyond the scope of this article (for reviews, see Hostetter, 2011; Kendon, 1994). Of interest to the current discussion is the role of gesture in multimodal communication, and how much information is transmitted through gesture in communication. Generally speaking, research has shown that gesture, in the presence of speech, has a communicative function (Hostetter, 2011; Kendon, 2004). Indeed, in some contexts, this aspect of the manual modality has been shown to carry 50-70% of the information in the overall message (Chovil, 1992). Comprehension of a message is facilitated and improved when gesture and speech (i.e., the vocal modality) are presented together (Holler et al., 2017; Holler & Wilkin, 2009; Kelly et al., 2010, 2015). This has also been shown in a more naturalistic face-to-face communicative setting (Holler et al., 2009). According to the integrated systems hypothesis, the integration of information from both channels happens automatically (Kelly et al., 2010, 2015). Such claims are further supported by studies that show a facilitating effect of gesture on word retrieval (e.g., Krauss et al., 2000; Murteira et al., 2018). How much interlocutors rely on gesture to produce or comprehend a message, however, depends on a number of factors, such as the type and complexity of information that is communicated, how concrete or abstract the information is, and whether the information is already present in speech or not (Hostetter, 2011). In addition, the assumption is often made that the degree to which a person can express or comprehend the entire message by relying solely on the vocal modality also influences how much gesture is relied on in communication. When language skills are non-optimal, such as in non-native speakers, in children, and in populations with language problems due to neurological or developmental impairments, it is often assumed that gesture can, in part, compensate for the loss in verbal abilities. A number of studies suggest that children’s comprehension and learning of complex concepts is better when gestures are combined with speech compared to when concepts are presented with just the vocal modality information (Ping & Goldin-Meadow, 2008; Singer & Goldin-Meadow, 2005; Wakefield et al., 2018). Veinott et al. (1999) showed that non-native speakers who could not use their language channel optimally due to a lack of proficiency benefited from the use of other communicative channels in communication, such as gesture, to supplement their comprehension.

##### **People with Aphasia**

There is a growing body of research on gesture in aphasia (for a review, see Rose, 2006; Rose et al., 2013b). Most of the research on gesture has focused on non-fluent aphasia, with a smaller number of studies that have evaluated gesture in those with fluent aphasia (for example, see Carlomagno et al., 2013). Overall, research has shown that people with aphasia produce gesture in communication. Some research suggests that people with aphasia produce more gestures compared to non-brain damaged controls (Carlomagno et al., 2005; Rousseaux et al., 2010; Sekine & Rose, 2013), but that they differ in the types of gestures produced in spontaneous speech (Sekine & Rose, 2013; van Nispen et al., 2016). People with aphasia who had a relatively intact conceptual system, as typically seen in non-fluent aphasia, were found to produce more meaningful gestures (Sekine & Rose, 2013), whereas those with a more fluent aphasia used more abstract and unspecified gestures (Cicone et al., 1979; Sekine & Rose, 2013). People with aphasia who had less severe linguistic impairments, such as in anomic aphasia, produced types of gestures comparable to controls. Although the number of studies is small, based on finding such as these, a number of researchers have proposed that gesture and language rely on the same underlying system and break down together in aphasia (for a brief discussion, see Cicone et al., 1979; Hogrefe et al., 2012).

In addition to looking at gesture production as such, researchers have also examined the communicative effectiveness of gesture in aphasia. These studies show that gestures can add communicative value to the message conveyed by people with aphasia in speech (de Beer et al., 2017; Hermann et al., 1989; Hogrefe et al., 2013; Mol et al., 2013; Rose et al., 2017). One study showed that on average, between 22-92% of gestures produced by people with aphasia were essential for understanding their message, as compared to 5% for controls (van Nispen et al., 2017). These essential gestures conveyed information in the absence of speech, added information that was missing in speech or helped clarify information presented in speech (Dipper et al., 2015; van Nispen et al., 2017). These findings argue against the simultaneous breakdown of gesture and language, as gesture compensates for loss of meaning in the lexico-syntactic channel. Therapy studies have shown that the use or comprehension of gesture can improve after gesture training (Daumüller & Goldenberg, 2010; Marshall et al., 2012; 2013; Roper et al., 2016). Effects from such interventions were shown on communication measures, such as the ability to convey a single message (e.g., “I took two pills”) and the ability to convey a sequence of ten linked events based on a silent video (Caute et al., 2013). Many of the studies on gesture production employ decontextualized gesture elicitation methods without the interactive, co-constructive nature of face-to-face communication. As different communicative situations may elicit different gesture behaviours (Hogrefe et al., 2012), it remains unclear whether the abovementioned results can be generalised to face-to-face communication. Rose et al. (2017) suggested that the lack of ecological validity in these studies may underestimate the communicative effectiveness of gesture in aphasic speech. Their study of spontaneously produced pantomime gestures in conversational discourse showed that speech and gesture combined had a strong communicative effect in aphasia (Rose et al., 2017). In a semi-structured conversational setting, even people with severe aphasia were shown to compensate for their impairment in the vocal modality by producing meaningful gestures (Hermann et al., 1989; Rose & Douglas, 2003). The same was found in a smaller study of spontaneous conversation between parrticipants with severe aphasia and a friend (Hermann et al., 1988). Importantly, a number of studies have suggested gesture production can be influenced by two factors that frequently co-occur with aphasia, namely, the presence of limb apraxia and impaired semantic processing (Cocks et al., 2013; Fucetola et al., 2006; Hogrefe et al., 2012; van Nispen et al., 2016). Limb apraxia is a motor planning disorder that affects the performance of purposeful movements (Gonzalez Rothi et al., 1991). Limb apraxia often co-occurs with aphasia and is said to affect the ability of people with aphasia to produce gestures such as pantomime (e.g., Hogrefe et al., 2012; van Nispen et al., 2016). Limb apraxia and semantic processing have been shown to be strongly interconnected (van Nispen et al., 2016). People with limb apraxia have been shown to have deficits in semantic processing that affect their ability to use pantomime gestures, for example, the ability to accurately select distinctive features from semantic representations. The exact relationship between aphasia, limb apraxia, and the use of gestures remains unclear (Goldenberg et al., 2003; van Nispen et al., 2016). Overall, it seems that gesture plays an important role in communication in aphasia and there is evidence that people with aphasia use gesture to complement and supplement their output through the vocal modality (de Beer et al., 2017; Rose, 2006).

Much less research has been done on gesture comprehension in aphasia. A number of studies have suggested that gesture comprehension is impaired in aphasia (Gianotti & Lemmo, 1976; Rousseaux et al., 2010), and that comprehension difficulties are more frequent in people with aphasia with semantic processing difficulties and in people with aphasia with posterior lesions compared to those with anterior lesions (Cocks et al., 2009; Daniloff et al., 1986; Ferro et al., 1980; Gianotti & Lemmo, 1976). Non-fluent aphasia, in turn, has been related to unimpaired gesture comprehension (Rose, 2006). As in production, gesture comprehension is also said to be affected by the presence of limb apraxia (Eggenberger et al., 2016).

A few small studies have assessed the added value of observing multiple channels, such as gesture and the vocal modality, in comprehension. Results have shown that adding gesture to the vocal modality can lead to improvements in comprehension in aphasia (Cocks et al., 2009; Eggenberger et al., 2016; Yorkston et al., 1979). Interestingly, it is still unclear whether people with aphasia benefit from the presentation of multiple channels of information by integrating the available information (multimodal gain; Eggenberger et al., 2016; Yorkston et al., 1979), or by relying on a single, possibly less impaired channel such as gesture (Cocks et al., 2009, 2018). Records (1994), for example, showed that as ambiguity increased in the vocal modality, people with aphasia relied more heavily on gesture (pointing behaviour) to construe meaning. Cocks et al. (2009, 2018) hypothesized that the lack of multimodal gain observed in their study could be caused by an impaired allocation, or reduced availability, of attentional resources, which prevents people with aphasia from processing all the available information. When gesture and the vocal modality provide congruent information, however, it seems possible for gesture to contribute to improved comprehension for people with aphasia, either by contributing to a multi-channel message or by offering an alternative channel to rely on in communication.

#### Face and Eye Movements

##### Neurologically Healthy Controls

In neurologically healthy controls, it has been shown that people monitor each other’s faces closely during conversation. Interlocutors gather information from facial movements (Bavelas & Chovil, 2000; Clark, 1996; Ekman, 1979; 1997), eye gaze (Goodwin, 1981; Hanna & Brennan, 2007; Kendon, 1967), lip movements (McGurk & MacDonald, 1976), and eyebrow movements (Flecha-García, 2010) to inform communication. Much research has been done on facial expressions and how they convey an underlying emotional state of a person (Parkinson , 2005). In interaction, facial expressions or facial movements can serve a communicative function on their own, or in combination with other channels of expression such as lexico-syntactic information (Chovil, 1997; Frith, 2009). It is assumed that facial expression can be used to efficiently communicate on a wide variety of topics, including emotions (Chovil, 1997), and to indicate levels of speaker certainty (Dijkstra et al., 2013; Swerts & Krahmer, 2005). Facial expressions can be used for linguistic purposes such as marking emphasis (Birdwhistell, 1970), indicating understanding, dislike, confusion, and disbelief, or difficulty in recalling an event (Chovil, 1992; Ekman, 1979). Eyebrow movements have been related to structuring and emphasising information in a verbal message (Ekman, 1979; Flecha-García, 2010). Smiles, along with nods and verbal expressions such as ‘yeah’, have been shown to function as backchannels to indicate continued attention and involvement in conversation, to signal the listener’s level of understanding and level of agreement (Brunner, 1979). Many of these facial movements are argued to only be interpretable in their conversational context, and not otherwise interpretable (Chovil, 1997). Furthermore, research has shown that gaze plays an important role in coordinating face-to-face communication (Bavelas et al., 2002; Hanna & Brennan, 2007; Kendon, 1967), for example, by regulating turn exchanges (Bavelas et al., 2002; Goodwin, 1981; Kendon, 1967). Gaze can also be relied upon as an indication of continued attention and for the direction of attention (Argyle & Cook, 1976; Emery, 2000; Goodwin, 1981; Itier & Batty, 2009). Speakers use gaze to monitor listeners’ understanding (Kendon, 1967), to seek and elicit a response and feedback (Bavelas et al., 2002; Rossano, 2013), to resolve temporal ambiguity in conversation (Hanna & Brennan, 2007), to emphasize or reinforce a verbal message, and to monitor conversation for possible difficulties (Emery, 2000). Gaze has been shown to combine with other cues, such as in the vocal modality and other signals from the face, in complex ways to create a composite message (Argyle & Cook, 1976). Visual cues from lip movements have been shown to help listeners anticipate what auditory information is coming, such as in the case of auditory and visual incongruencies (McGurk & MacDonald, 1976), or when there is noise in the auditory signal (e.g., Jordan & Sergeant, 2000).

A different line of research has assessed the effect of visibility of the conversation partner’s face on the efficiency of communication. Rather than focusing on specific elements of the face, these studies assess the effect of being able to see the face of the other speaker compared to not being able to see the face at all, thus exploring the combined effect of the elements discussed above. A number of studies have shown that efficiency on a collaborative task is heightened when interlocutors can use the visual channel in communication (Boyle et al., 1994). In one study by Boyle et al. (1994), efficiency was measured by the total time and number of turns it took to complete the task. Overall performance was not affected in this study, meaning that participants could still complete the task successfully without the use of the visual channel, but the task took longer and required more turn exchanges between the interlocutors. Critically, the extent of reliance on signals from a conversation partner’s face seems to depend on the task. Lysander and Horton (2012) and Clark and Krych (2004) found no facilitative effect of mutual visibility on collaborative card-matching and lego-building tasks, respectively. Instead, communication efficiency depended on having a shared view of the task-relevant materials, being the objects both interlocutors were referring to. This effect is further discussed in the “Common Ground”. Lysander and Horton (2012) argued that the lack of effect of mutual visibility on task efficiency was likely to have been caused by the need to attend to the stimuli. In addition, it seemed the neurologically healthy controls in their study were able to solve communicative difficulties through other modalities, such as vocalization. These neurologically healthy controls may not have needed additional information from another channel or modality to understand their interlocutor. In short, during communication, a large amount of information can be conveyed through the face independently, or combined with other channels such as gesture and lexico-syntax.

##### People with Aphasia

In production, people with aphasia have been shown to use facial movements in interactions to show emotions (Laakso, 2014a), and to indicate problems in conversation, such as with eyebrow movement, smiling, and laughter (Kaukomaa et al., 2014; Laakso, 2014a). Goodwin (1995) provided a detailed description of how a man with severe aphasia used eye gaze to inform his conversation partner of his attentiveness to what was said, as well as to demonstrate his departure from listening by diverting his gaze. More generally speaking, people with aphasia with left hemisphere lesions are often assumed to have intact pragmatic abilities in communication, which would include face and eye-movements as described above. This fits with our review of studies on backchannels and feedback in conversation (see section *Language use is interactive*), which found that these skills are often largely intact for people with aphasia.

Very little research has explored the use of visual information from the face by people with aphasia to aid communication. A few studies have suggested that people with aphasia may have difficulty integrating information from the visual and vocal modalities (Preisig et al., 2015; Schmid & Ziegler, 2006; Youse et al., 2004). This is line with the claim that people with aphasia may not be able to benefit from multimodal stimuli in their comprehension of gesture (Eggenberger et al., 2016). Preisig et al. (2015) suggested that the impairment of the vocal modality interferes with the integration of the vocal signal with the available visual information. According to Preisig et al. (2015), people with aphasia then rely on the signal that carries the most information, rather than on a combination of the two. For example, these researchers showed that during co-speech gesture, people with aphasia exhibited similar fixation patterns on the speaker’s hands compared to neurologically healthy controls when observing natural dyadic conversation. Interestingly, independent of co-speech gesture, people with aphasia showed a reduced fixation on the speaker’s face. This could indicate that people with aphasia did not, or could not, compensate for their difficulty in comprehension of information in the vocal modality by focusing on visual cues from the face. In their case-study, Youse et al. (2004) showed people with aphasia did not benefit from a bi-modal presentation (visual and auditory), compared to a unimodal condition (auditory), on a speech perception task. This supports the claim that people with aphasia have difficulty integrating information from different channels and modalities (also seen in the literature on gesture comprehension, see above). From the literature, it remains unclear whether or not people with aphasia rely on visual information from their conversation partner’s face to better comprehend their message.

#### Prosody

##### **Neurologically Healthy Controls**

In addition to the content of the verbal message (*what we say*, i.e., the lexico-syntactic information) we can convey information by changing the way in which we say something (i.e., prosody). Prosody refers to variations in speaking rate, pitch, loudness, and voice quality, which each play a part in conveying meaning (Hellbernd & Sammler, 2016). For neurologically healthy controls, prosody can change the meaning of a message at a linguistic and a paralinguistic level (Bolinger, 1986, for reviews, see Cole, 2015; Cutler et al., 1997; Hellbernd & Sammler, 2016; Wagner & Watson, 2010). At a linguistic level, prosody can express semantic relationships, disambiguate syntactic structures (Cutler et al., 1997; Wagner & Watson, 2010), group words into phrases (Wagner & Watson, 2010), signal the relative prominence or importance of a word, and signal illocutionary force, for example by marking an utterance as a statement or a question (Cole, 2015; Eberhard et al., 1995; Wagner & Watson, 2010; Witteman et al., 2011). Paralinguistically, prosody conveys information regarding the emotional state of the speaker (Cole, 2015; Scherer, 1986), as well as speaker certainty, confidence, and doubt (Jiang & Pell, 2017; Swerts & Krahmer, 2005), and speaker attitude and beliefs (Bolinger, 1986; Ladd, 1996). Prosody has also been found to play a role in managing interactions, also referred to as conversational prosody, for example, managing turn changes (Ford & Thompson, 1996; Selting, 2005), signalling the end of a turn (Bögels & Torreira, 2015), marking a new topic, expressing agreement with the interlocutor, expressing intentions, and facilitating the flow of discourse through pitch variation in backchannels such as “mm-hm”, “okay” and “yeah” (Cole, 2015; Hellbernd & Sammler, 2016; Wennerstrom, 2001).

In face-to-face communication, prosody interacts with other communicative channels such as facial expressions (i.e., smiles, head nods, eyebrow movements, and eye gaze) to convey meaning (Cole, 2015; Dijkstra et al., 2013; Flecha-García, 2010; Kendon, 1980; McNeill, 1992; Swerts & Krahmer, 2005). Across speakers, there is much variation in the use of prosody. The meaning of a prosodic cue is interpreted within the context of a particular syntactic structure, a discourse environment, and in the context of a particular speaker (Cole, 2015; Hirschberg, 2002). Importantly, however, prosody is used by interlocutors to improve comprehension, and plays a role in building meaning in face-to-face communication (Cole, 2015; Hellbernd & Sammler, 2016). Research has shown that to guide turn projection during conversation, such as when interlocutors expect a turn to end, adults and children benefit most from having both lexico-syntactic and prosodic information (when this information is congruent, providing multimodal gain). When lexico-syntactic and prosodic information is contrasting, lexico-syntactic information has been shown to be weighed more heavily (Lammertink et al., 2015). Based on their research with neurologically healthy controls in children and adults, Lammertink et al. (2015) suggest that to fully benefit from prosodic information for turn structure in conversation, some lexico-syntactic information is necessary. This idea is supported by other research (Casillas & Frank, 2017; Männel & Friederici, 2010). Finally, there are cross-cultural differences in the interpretation of, and reliance on, facial expression and intonational differences in conversation (Crespo Sendra et al., 2013). This suggests that findings about multimodal integration, and the weight assigned to particular channels in one language, may not translate to other languages or cultures. Consequently, the same language impairment may affect communication differently depending on the weights that particular languages and cultures assign to each channel. These possibilities underscore the need for cross-cultural research on communication and language impairments.

##### **People with Aphasia**

Though prosody has a communicative function, it remains relatively understudied in aphasia (for a review of the literature, see Geigenberger & Ziegler, 2001). Much research has focused on the hemispheric specialization of different prosodic features (Witteman et al., 2011), emphasizing the difference between right and left hemisphere lesions. Often, these studies do not specify whether or not participants with left-hemisphere lesions include those with a diagnosis of aphasia. In the aphasiology literature, more research has been done on the role of prosody in production compared to comprehension. Even less work has been done on the contribution of prosody to comprehension in conversation. A number of studies have attempted to describe the different characteristics of prosody in the production of people with fluent and non-fluent aphasia. Generally speaking, some aspects of prosody have been shown to be relatively intact, although this varies across types of aphasia. Aspects such as fundamental frequency (F0) and timing in prosody have been shown to deviate in people with aphasia compared to neurologically healthy controls (Beeke et al., 2009; Danly & Shapiro, 1982; Danly et al., 1983; Rhys et al., 2013; Seddoh, 2000). The question that is most relevant for the current discussion is how much prosody contributes to communicative effectiveness in aphasia, in both comprehension and production. At the moment, the answer to this question is unclear. Different approaches have been used in the literature to attempt to answer this question. Walker et al. (2009) showed that people with aphasia produced prosodic structures that were different to those produced by controls on a word and sentence production task. Crucially, identifying the intended meaning in those utterances was more challenging for naive listeners for items produced by people with aphasia compared to those produced by controls. A number of studies have reported the compensatory use of prosody by people with aphasia in communication. By combining limited verbal output (i.e., lexico-syntactic information), ranging from a few words to lexically empty syllables with variations in pitch and volume, non-fluent people with aphasia have been shown to convey meaning. In line with the evidence we saw for the production of backchannels, people with aphasia have been shown to signal a demand for attention, call a listener, express uncertainty, agreement, enthusiasm, or appreciation, manage interaction and turn-taking, and finally request for something to happen, such as for someone to continue or stop guessing, or for the provision of information (Beeke et al., 2007, 2009; Goodwin, 1995, 2000; Lind, 2007; Oelschlaeger & Damico, 1998; Rhys et al., 2013). Dogil et al. (1990) presented a case study of a person with fluent aphasia who compensated for his language impairment by using unimpaired prosodic skills effectively. Despite the fact that his speech largely contained incomprehensible jargon, the person with aphasia was able to rely on prosody to express communicative intent clearly, including the expression of emotion and grammatical structures in discourse. During communication, the person with aphasia was able to handle discourse tasks such as topic change, expressing disagreement, and posing various types of questions effectively through prosody. Naïve, neurologically unimpaired participants were able to identify these discourse tasks reliably based on an audio recording of the person with aphasia’s speech, but not when using a transcript of the verbal, lexico-syntactic output.

A small number of studies have examined the comprehension of prosody in aphasia. Emotional prosody, for example, on a prosody and facial expression matching task, is suggested to be relatively unimpaired in aphasia (Barrett et al., 1999; Geigenberger & Ziegler, 2001; Pell & Baum , 1997; Perlman Lorch et al., 1998), although the opposite has also been reported (Pell & Baum, 1997; Pell, 1998). Linguistic prosodic processing has been shown to be impaired in aphasia, such as the ability to recognise focus or emphasis on prominent entities in an utterance (Geigenberger & Ziegler, 2001; Baum, 1998), and the ability to indicate whether a sentence is a statement or a question (Pell & Baum, 1997; Perkins et al., 1996; Seddoh, 2006). Pashek and Brookshire (1982) and Kimelman and McNeil (1987) showed that the use of emphatic stress can facilitate comprehension of spoken language for some people with aphasia. Pell and Baum (1997) showed that prosody recognition by people with aphasia was impaired on linguistic stimuli that required processing of syntactic or semantic cues, as well as prosodic cues, simultaneously. The authors argued that the processing of multiple linguistic cues might have been beyond the person with aphasia’s cognitive capacity (Pell & Baum, 1997). Note that this finding is in line with the research on gesture, which showed difficulty when multiple sources of information needed to be processed (Cocks et al., 2009, 2018).

Importantly, the aforementioned studies assessed the comprehension of prosodic structures in aphasia in a decontextualized and non-interactive setting. A different approach to assess the role of prosody in comprehension was taken by researchers who studied eye movements of people with aphasia, who in turn observed spontaneous, dyadic conversations. Healthy controls were shown to shift their eye gaze in anticipation of a change in turn, which is commonly predicted by lexico-syntactic information and prosody. As lexico-syntactic information increased, healthy controls were shown to benefit more from variation in intonation in predicting upcoming turns. People with aphasia did not show this reliance on intonation cues (Preisig et al., 2016), suggesting that perhaps people with aphasia cannot rely on linguistic prosody, or are unable to integrate information from the two channels. In conclusion, there is some support for the idea that people with aphasia can utilise prosody in production to communicate effectively, with most of the support for this claim coming from observational research with people with non-fluent aphasia. Whether or not people with aphasia can use prosody to support comprehension in conversation is unclear. The findings so far indicate that the integration of information from multiple channels may be more difficult for people with aphasia, or that people with aphasia may have difficulty flexibly changing the weight they give to different sources of information in order to resolve ambiguity or complexity (e.g., using prosody to aid comprehension of syntactically complex sentences; Preisig et al., 2016).

Although the use of a number of different channels has been studied in aphasia, these studies are limited in their generalisability to face-to-face communication because they have been studied in isolation from other channels in a non-interactive setting. Systematic analyses investigating the advantages of communicating through multiple channels (vocal and visual) should be conducted in an interactive setting to better understand how multimodal communication is affected in people with aphasia.

### Common Ground

Research with neurologically healthy controls shows that interlocutors use what has been said (communicative context), what is physically present (situational context), and shared experiences (personal and communal common ground) to guide how they produce and understand language during conversation, for example, by using more detailed descriptions when speaking to someone who doesn’t share a particular piece of knowledge (Brown-Schmidt et al., 2015; Brown-Schmidt & Hanna, 2011; Hanna et al., 2003; Heller et al., 2012; Schober & Brennan, 2003). There is ongoing debate regarding the cognitive mechanisms that underpin common ground during communication, whether there is active tracking of what is shared knowledge between two speakers, or whether the interpretation of references is made based on domain-general systems such as memory, with a limited role for speaker-perspective-based interpretations (Kronmüller & Barr, 2015). For clarity, research on each type of common ground will be discussed separately below.

#### Communal and Personal Common Ground

##### **Neurologically Healthy Controls**

The effect of having shared past experiences, beliefs, and knowledge with another interlocutor (i.e., communal or personal common ground) in face-to-face communication shows that more common ground can indeed lead to more efficient communication. Research with neurologically healthy controls has shown that familiar interlocutors use more abbreviated and informal language compared to unfamiliar pairs, relying on shared knowledge and experience during communication (Clark, 1996; Herrmann, 1983; Hornstein, 1985). Similarly, unfamiliar conversation partners have been shown to use more gestures compared to familiar conversation partners (Kistner, 2017), possibly reflecting the tendency to be more explicit and elaborate to avoid misunderstandings with an unfamiliar conversation partner. On the other hand, familiar conversation partners have been shown to initiate more topics, ask more questions, and provide more minimal turns during conversation compared to strangers (Boyle et al., 1994; Hornstein, 1985). During a collaborative task, Boyle et al. (1994) showed that despite the increase in number of turns and words, familiar pairs showed more efficient communication with fewer interruptions and overlaps in speech. With a familiar partner, the person following instructions was found to look at the instructor more often than with an unfamiliar partner. This suggests that familiar partners might use visual cues to support communication more than unfamiliar conversation partners. Overall, the findings from Boyle, Anderson, and Newlands’ (1994) study suggests that familiar pairs are better at interpretating auditory or verbal, visual, and paralinguistic cues from familiar partners compared to unfamiliar interlocutors, due to having existing shared experiences, knowledge, and beliefs (Herrmann, 1983).

##### People with Aphasia

Although it remains largely unknown *how* the familiarity of a conversation partner affects communicative efficiency for people with aphasia, it is generally believed that it does affect communication for people with aphasia (Ferguson, 1994, 1998; Green, 1982; Howe et al., 2008; Laakso & Godt, 2016; Perkins, 1995; Wirz et al., 1990). Questionnaires on communication often distinguish between familiar and unfamiliar conversation partners (e.g., the disability questionnaire of the Comprehensive Aphasia Test, Howard et al., 2004). Interestingly, a recent study by Leaman and Edmonds (2019) compared conversations between people with aphasia and a familiar conversation partner to conversations with an unfamiliar partner with a speech and language therapist. The researchers found that the small sample of people who mostly had mild anomic aphasia showed no significant differences on measures of sentence production (such as the sentence frame, relevance of the lexical items, and morphological and verb tense or mood errors), nor in the overall judgement of communicative success (Leaman & Edmonds, 2019). These findings suggest that some elements of conversation remain stable across different conversation partners (Leaman & Edmonds, 2019). Kistner (2017) showed that neurologically healthy controls and people with aphasia used more gestures when speaking to an unfamiliar conversation partner compared to a familiar speaker. This may reflect the fact that when speaking to an unfamiliar person, one cannot rely on implicit, abbreviated, and informal language and thus more elaborate and explicit language and gestures are used.

#### Communicative Context

##### **Neurologically Healthy Controls**

Speakers and listeners rely on the communicative context, being what has already been said or communicated during conversation, to guide their own production and comprehension during an interaction. For example, the production of certain words and sentences by one interlocutor can influence the selection of words and sentence constructions by another. Speakers tend to express themselves in similar ways at the lexical, semantic, and syntactic level (Branigan et al., 2000). Priming studies have shown that speakers tend to implicitly produce sentences and lexical items that are similar to those produced by their conversation partner (Bock et al., 2007; Branigan et al., 2000; Mahowald et al., 2016). When participants work on a collaborative task, they converge on specific descriptions (Branigan et al., 2000) and lexical expressions that refer to particular stimuli (Clark & Wilkes-Gibbs, 1986). Neurologically healthy controls have also been shown to flexibly and successfully rephrase and restate the speech of others or themselves during conversation, referred to as reported speech (Hengst et al., 2005; Myers, 1999). This makes language production computationally less taxing, as the choices for word or sentence structure are “to a considerable extent driven by the context and do not need to be a burden for the speaker” (Pickering & Garrod, 2004, p. 15). When interlocutors work with the same stimuli on a collaborative task, they tend to use increasingly similar expressions to refer to stimuli, in which they progressively use fewer words, require fewer turns, and provide less content (Brennan & Clark, 1996; Clark & Wilkes-Gibbs, 1986; Fussell & Krauss, 1992; Garrod & Anderson, 1987; Horton & Gerrig, 2005; Isaacs & Clark, 1987; Schober, 1993; ). An example of this is the reference to a tangram figure which developed from ‘a person who’s ice skating, except they’re sticking two arms out in front’ to ‘the person ice skating, with two arms’ to ‘the ice skater’ (Clark & Wilkes-Gibbs, 1986). The same effect has been found for gestures. Gestures became less complex, less informative, less precise, and less elaborate when they were directed at an interlocutor with shared knowledge on the task (Gerwing & Bavelas, 2004; Holler & Stevens, 2007; Mondada, 2007). As the stimuli become part of common ground, it seems interlocutors can exert decreasing effort to refer to the same entities.

Crucially, common ground is constructed uniquely by two conversation partners. When, halfway through the task, one of the partners is replaced, a decrease in number of words, turns, and content seen with the initial partner is reversed. In other words, efficiency decreases when the communication partner is changed during a communicative task (Brennan & Clark, 1996; Schober & Clark , 1989; Wilkes-Gibbs & Clark, 1992). In addition, listeners are slower to respond to the same established reference from a new speaker compared to the same utterance provided by the speaker who established the reference in the first place (Metzing & Brennan, 2003). In fact, repeated use of the same referring expressions is expected by listeners (Barr & Keysar, 2002; Shintel & Keysar, 2007). Listeners show surprise when speakers change their referring expression (Metzing & Brennan, 2003), or ask questions to ensure the same entity is targeted (Garrod & Anderson, 1987). This is in line with research that shows that listeners build up expectations about what is to come based on what they have heard so far (Skipper, 2014). Similarly, research has repeatedly shown that the context of a sentence or a gesture restricts the number of possible expected meanings of a word (Griffin & Bock, 1998; Kutas & Federmeier, 2011; Piai et al., 2014; Skipper, 2014). This effect has also been shown in text and discourse (for a review, see van Berkum, 2009), supporting the idea that the language system integrates word, sentence, discourse, and common ground information to allow listeners to predict and interpret language (e.g., Barr & Keysar, 2002; Kutas & Federmeier, 2011; MacDonald et al., 1994). Information that is part of discourse also allows for the use of shorter references (e.g., Barr & Keysar, 2002; MacDonald et al., 1994), and the recent discourse history helps interlocutors resolve semantic-pragmatic ambiguities. For example, the interpretation of anomalous sentences such as ‘the girl comforted the clock’ may depend on fitting within a particular context (Nieuwland & Berkum, 2006).

In short, reliance on the communicative context allows interlocutors to minimize the efforts made in production. Speakers can use shorter and less complex utterances by coordinating lexical and syntactic structures with their conversation partner, and by relying on the ‘givenness’ of information during face-to-face communication. Comprehension can also be facilitated by the communicative context, as it restricts the number of possible interpretations a word or expression can have, and allows listeners to predict what will be communicated next.

##### People with Aphasia

In aphasia, less experimental research has been done on the use of common ground in communication. Although the use of the communicative context in interaction has not been studied extensively, there is evidence to support the idea that people with aphasia benefit from communicative context in production and comprehension (e.g., Pierce, 1991). Similar to non-brain damaged controls, people with aphasia have shown responsiveness to priming effects at the lexical level, for example, hearing or reading a word can make it easier to produce a semantically related or identical target word in picture naming tasks (Cornelissen et al., 2003; Love & Webb, 1977; Renvall et al., 2003; Renvall et al., 2007). A slightly different facilitatory effect was shown by McCarthy et al. (2017). Impaired repetition of abstract words (e.g., ‘distance’) was facilitated by training its repetition in an enriched semantic environment. The enriched semantic environment was created by coupling the abstract word with a more accessible highly-imageable, high-frequency word (e.g., ‘long’, resulting in ‘long distance’). The presence of a semantically constraining sentence has been shown to facilitate lexical retrieval on a picture-naming task (e.g., Piai et al., 2017). Responsiveness to this kind of context has been shown to depend on the lesion location (Piai et al., 2017) and the nature of the impairment. If the underlying impairment is more phonological in nature, contextual phonological cues will be more beneficial. If the impairment is more semantic in nature, semantic cues will have a more facilitatory effect (Martin & Laine, 2000; Martin et al., 2004). Similar priming effects have been shown for syntactic structures. People with aphasia were increasingly likely to produce specific syntactic structures after hearing similar syntax during a picture description task (Cho-Reyes et al., 2016; Hartsuiker & Kolk, 1998; Rossi, 2015; Saffran & Martin, 1997). In addition, sentence-level interventions based on priming mechanisms have been shown to improve picture description sentences in people with aphasia (Lee & Man, 2017; Mack et al., 2017; Weinrich et al., 2001). Finally, in an exploratory study Pashek and Tompkins (2002) showed that for both participants with mild anomic aphasia and neurologically healthy controls, the linguistic context facilitated lexical retrieval in connected speech using a video narration task as compared to a confrontation naming task. This suggests that communicative context may positively influence language production for people with aphasia. This effect, however, may differ between aphasia syndromes such as fluent compared to non-fluent aphasia (Richardson et al., 2018; Williams & Canter, 1982).

A small number of studies have suggested that reported speech, such as the conscious repeating of output from a conversation partner to produce a similar syntactic structure or lexical item, is used by some people with aphasia in everyday interactions (Hengst et al., 2005). Despite the high number of errors and failed attempts to repeat complete utterances, reported speech has been shown to contribute to successful communication in aphasia. In a case study, Oelschlaeger and Damico (1998) showed that the explicit repetition of the conversation partner’s utterances enabled one person with aphasia to achieve conversational goals, such as expressing agreement and uncertainty, that would otherwise not have been possible due to his very limited spontaneous verbal abilities.

Like neurologically healthy controls, people with aphasia have been shown to use increasingly fewer turns and shorter, more simplified references during a collaborative referencing task with familiar conversation partners (Hengst, 2003; Meuse & Marquardt, 1985). This supports the idea that people with aphasia can rely on common ground and produce increasingly shorter, less complex utterances to refer to ‘given’ information during an interaction.

Finally, the presence of a communicative context has also been shown to support comprehension in aphasia (Dickey et al., 2014; Pierce, 1991; Warren et al., 2016), although this facilitative effect is slower compared to non-brain damaged controls. Similarly, people with aphasia showed an N400 effect similar to that of neurologically healthy controls when hearing a semantically unexpected word in a sentence, however this effect is less pronounced and delayed in people with aphasia (Hagoort et al., 1996; Khachatryan et al., 2017; Swaab et al., 1997). people with aphasia were also able to predict upcoming syntactic structures based on the context of the sentence (Hanne et al., 2015a, b). Having a communicative context that limits the number of possible meanings of an utterance can thus alleviate some of the processing demands involved in comprehension (Pierce, 1991). It is not known whether people with aphasia can benefit from this type of context during face-to-face communication, especially given the time-pressures of real-world communication and the potential delay in processing observed in the above-mentioned studies. Conversation analysis on this process has shown that, in a more general sense, people with aphasia use the communicative, *sequential* context as a resource to construct their turns during conversation, and to aid their comprehension of what others are communicating (Beeke et al., 2007).

#### Situational Context

##### Neurologically Healthy Controls

The physical environment, or the referential situation, is used to support production and comprehension during face-to-face communication (Knoeferle & Guerra, 2016). Lysander and Horton (2012) and Clark and Krych (2004) showed that the communicative efficiency of participants depended on the shared view of the task-relevant materials. Overall, communication was more efficient when the materials that were discussed  were visually and referentially available to both participants compared to when they were not. In production, research has shown that neurologically healthy controls monitor their surroundings for non-linguistic ambiguity before speaking to ensure their utterance is informative in the current environment (Rabagliati & Robertson, 2017). Speakers thus adapt their expressions during communication based on the visual availability of the objects they are describing to their conversation partners. For example, if only one out of two buckets is visually available to a listener, speakers have been shown to use a less specific description such as ‘the bucket’, compared to when both objects are visually available to the listener. The utterance then includes more detail to specify, for the listener, which bucket is referred to (i.e., ‘the small bucket’, Brown-Schmidt & Duff, 2016; Yoon et al., 2012). Further support for the reliance on perceptually available information also comes from developmental research and studies of second language learning. Between the ages of two and four, children have been shown to develop the ability to change their referring expressions based on the availability of information in discourse and the perceptual availability of the referents for their conversation partners (Matthews et al., 2006; Moll & Tomasello, 2006; Salomo et al., 2011). Furthermore, children and second language learners have been shown to acquire the ability to refer to objects and events not currently present (described as displaced reference), later than the ability to refer to the here-and-now (Sachs, 1983). Research in second language learning has supported the idea that displaced reference is more effortful than speaking about the here-and-now, and that displaced reference requires increased linguistic complexity (Gilabert, 2007; Ishikawa, 2007; Robinson & Gilabert, 2007a; Robinson, 1995). Indeed, when speaking about the here-and-now, people can point at, touch, exhibit, and present physical objects to support communication (Clark, 2005). Indirect evidence for this comes from research on the processing of concrete and abstract concepts. Research has shown that it is easier for neurologically healthy controls to produce and understand concrete concepts compared to abstract concepts (Evans et al., 2012; Paivio, 1991; Roxbury et al., 2014). Although concreteness is not synonymous to visual or physical availability, concrete concepts are more tangible, have a higher imageability (i.e., it is easier to generate a mental image), higher contextual availability (i.e., it is easier to retrieve relevant information about the concept from memory), and can be experienced through the senses (seeing, touching, etc.). In contrast, abstract concepts are less tangible, have lower imageability, lower contextual availability, and are often less experienced through the senses because often they do not have real-world referents (Paivio, 1986; Schwanenflugel et al., 1992). According to Zwaan (2014), abstract concepts require the involvement of more long-term memory processes and increased reliance on language processing, both of which increase the use of cognitive resources.

Research has also provided support for the idea that the visual environment can affect and restrict the way (ambiguous) linguistic input is interpreted (Chambers et al., 2002; Eberhard et al., 1995; Huettig et al., 2011; Tanenhaus et al., 1995), and that it can help predict what linguistic information is coming next (Huettig et al., 2011; Skipper, 2014). In one study, memory-impaired patients with hippocampal amnesia who could not rely on information stored in memory (common ground) to resolve linguistic ambiguities were shown to use visual information to guide behaviour (Rubin et al., 2011). The presence of referents in the visual environment can aid comprehension by limiting the possible interpretations of linguistic information, thereby reducing cognitive load during comprehension.

##### People with Aphasia

In clinical practice, people with aphasia are trained to compensate for their language loss by, if possible, pointing to objects in the physical environment to support communication. An observational study by Howe et al. (2008) indicated that the availability of a physical referent in the environment can facilitate communication in real world settings. Visual information in the form of relevant, contextualized photographs, or a television program depicting a specific setting with clear situations, places, experiences, and people, have been shown to facilitate reading comprehension (Dietz et al., 2013), comprehension of spoken language (Pierce & Beekman, 1985), as well as communication in aphasia (Howe et al., 2008). With the help of such aids, conversations can last longer, more content is exchanged, and the total number of exchanges increases (Beukelman et al., 2015; Garrett & Huth, 2002; Ho et al., 2005; Hux et al., 2010; Ulmer et al., 2016). The use of contextually rich photographs or videos is hypothesized to facilitate communication because it creates a shared communication space that includes content and background information, which the person with aphasia can refer to in support of comprehension and expression (Beukelman et al., 2015; Ho et al., 2005; Howe et al., 2008; Hux et al., 2010). The presence of a shared communication space through photographs is comparable to having a referential context during communication, in the sense that not all information has to be retrieved from memory or coded linguistically because it is visually available (Beukelman et al., 2015; Dietz et al., 2009). This may decrease the cognitive demands of the interaction (i.e., lessen the need for memory retrieval or complex linguistic structures, Zwaan, 2014). Howe et al. (2008) also suggested that the familiarity of a setting or particular physical environment can influence the ease with which a person with aphasia can rely on this information during communication. In line with the literature on non-brain damaged controls, research on aphasia has also shown that it is easier for people with aphasia to process concrete words compared to abstract words (Alyahya et al., 2018; Sandberg & Kiran, 2014). As discussed above, this provides indirect support for the idea that objects that are more likely to have real-world referents and thus may be pointed at, drawn, or more easily imagined, are easier to understand and name compared to objects that are less likely to have real-world referents. Interestingly, Davidson et al. (2003) showed that conversations of older people with aphasia tended to focus on topics in the ‘here-and-now’, which was not the case for older neurologically unimpaired controls. It remains to be investigated what underlies this tendency. In sum, there is evidence that some people with aphasia can use situational context (in the form of photographs, communication aids, and so on) to support their communication. An interesting question is whether this is universal for people with aphasia, or if there are individuals who find using such support more difficult, and if that is related to, for example, limitations in executive function, working memory, and attention. An extension of this question is also of interest, namely, whether the physical environment in which a conversation is held influences communication for people with aphasia, and whether this differs across individuals.

## Discussion

Language has often been studied without reference to the context in which it is used in the real world. Researchers have, over the past few decades, realized that this traditional approach does not allow us to understand the way language functions when it is used for communication in the real world. This is true for language research in general, as well as for aphasia rehabilitation specifically, where it is essential to measure functioning and intervention outcomes at the level of everyday communication. In light of the central importance of communication for aphasia rehabilitation, it is imperative that a more systematic and theoretically founded approach to the study of everyday language use is applied to the study of aphasia.

In this paper, we summarised a framework for situated language use, borrowed from the fields of communication sciences, psychology, linguistics, and sociology (see Table 1). The framework was used to review literature from neurologically healthy controls and people with aphasia, illustrating how language is used in situ, and how this behaviour differs from language processing in vacuo. Language in situ is (1) multimodal, (2) interactive, and (3) reliant on common ground. A number of conclusions can be drawn from the current review, and this definition of real-world communication as it is applied to aphasia rehabilitation.

### Interactivity

When considering the interactive nature of situated language use, there is a substantial and robust evidence-base for communication partner training. Communication with people with aphasia can be improved when conversation partners have an improved knowledge of aphasia and training in communication strategies. These approaches should be an essential element of aphasia therapy for all practitioners. There is evidence that the production and comprehension of backchannels and feedback is preserved for left-hemisphere stroke, that people with aphasia (at least those who are non-fluent) can self-monitor during communication, and that people with aphasia can be trained to produce more and better targeted feedback to manage the conversation with a specific partner. Further work is needed to explore how the use and understanding of feedback can be easily assessed, how it relates to an individual’s cognitive profile, communicative performance, and aphasia symptoms, and whether it is typically amenable to training and intervention.

### Multimodality

A wider body of research has investigated multimodal communication in aphasia, although it has typically separated different channels such as gesture, gaze, and prosody. There is substantial research on the role of gesture in aphasia, both in comprehension and production. Gesture has been shown to be an important part of the communication process for people with aphasia, and research shows that the use of gesture is different in real-world communication compared to in decontextualized tasks in the lab. The impairment of comprehension and production of gesture varies across aphasia types and severities. A number of intervention studies have provided support for the idea that gesture production and comprehension can be effectively trained through therapy. Although some research has been done to assess gesture use during real-world communication, more research is needed to fully understand its role in communication for people with aphasia, especially across different impairment profiles. The finding that gesture use and comprehension differs between decontextualized and more naturalistic settings highlights the need for more research on the latter.

A topic of interest should be the production and comprehension of multiple communicationn channels. It is not clear whether all people with aphasia benefit from the presence of multiple channels, or whether they are able to use the available information to their benefit during comprehension (i.e., multimodal gain). In our review of the literature, we found evidence of difficulty integrating or using multiple signals in work on gesture, prosody, and when language is presented in visual and vocal modalities. It may be that, during communication, people with aphasia become reliant on a single informative modality (e.g., using more visual information when comprehension of the vocal modality is impaired). There was also evidence to suggest that people with aphasia can benefit from information from multiple channels when the information is congruent, but not when it is incongruent. Incongruent or ambiguous information may require additional cognitive resources in order to resolve the conflict, and hence may be more difficult for some people with aphasia.

Difficulty with multimodality in comprehension and production is consistent with theories of aphasia that propose executive dysfunction as part of some profiles of comprehension impairment, with regard to difficulty identifying and using relevant cues (e.g., Thompson et al., 2015, 2018). This also fits with theories of aphasia that propose a limitation or reduction in available cognitive resources (see section *Cognition* below). The strategic use of multiple communication channels and the requirement to switch between them, or emphasize some over others, is one factor that likely makes some people with aphasia better communicators (Holland, 1977). In production, total communication, defined as using any and all available means of communicating, is often implemented as a strategy for people with moderate to severe aphasia who have more limited verbal output (Rautakoski, 2008; 2011a, b). We should be keen to understand how this skill is preserved or impaired in production, whether multimodal gain is observed during comprehension, and whether it can be trained.

### Common Ground

Healthy adults use pre-existing common ground, such as conversation partner familiarity, common ground that arises during a conversation, and the physical environment, to minimize the effort required for both production and comprehension during communication. For example, healthy adults coordinate their speech at the word and sentence level with their conversation partner(s) and rely on shared knowledge to use shorter and less complex utterances. There is evidence that some of these skills are preserved in aphasia, as people with aphasia use reported speech and the communicative and situational context to construct turns during conversation. We do not yet fully understand how well people with aphasia use perceptually available information and the physical environment to minimise effort during everyday communication. Low-tech augmentative and alternative communication, such as communication books and photographs, have been shown to facilitate communication for people with aphasia. However, there is no systematic understanding of when and how the communicative, physical, and situational context is used by people with aphasia during real-world communication.

### Cognition

Situated ‘natural’ language use clearly requires a range of skills to be deployed and co-ordinated, often simuleaneously (e.g., language comprehension, language production, discourse monitoring, social interaction, monitoring whether you have been understood, multimodal integration) and in real-time with a pressure to respond promptly (Meteyard, 2020). The framework therefore highlights the increased cognitive complexity of natural, situated language use compared to isolated language tasks, and the need to understand the role of such cognitive demands on communication for people with aphasia. While the cognitive complexity of real-world communication is well recognised in, for example, second-language learning (Robinson & Gilabert, 2007b), it is not yet properly understood in the field of aphasia rehabilitation.

It is highly likely that a situated language task places a greater burden on attention and working memory. Impairments that affect these cognitive resources have been shown to be important contributors to the success of aphasia rehabilitation (e.g., Salis et al., 2017). Research has shown that these cognitive resources are often reduced or impaired for people with aphasia, including executive functions such as cognitive flexibility, switching, and inhibitory control (Chiou & Kennedy, 2009; El Hachioui et al., 2014; Kendrick et al., 2019; Murray, 1999). Flexibility and the ability to switch, for example, using an alternative or additional channel of expression such as gesture during communication (e.g., Purdy & Wallace, 2015; Wallace & Kayode, 2016), are precisely the kinds of skills that will be important for successful situated language use in aphasia rehabilitation. Furthermore, impaired cognitive functions have been associated with particular symptoms experienced by people with aphasia and their ability to communicate in the real world. Impaired sustained and selective attention have been suggested to affect auditory comprehension and spoken language during communication, for example, when understanding longer chunks of information (Ferstl et al., 2005; Groenewold et al., 2014, Murray, 2012). Executive functions are said to be involved in (self-)monitoring during communication when different types of linguistic information such as semantics and syntax are integrated, and when relevant information has to be retained and manipulated during interaction (El Hachioui et al., 2014; Helm-Estabrooks, 2002).

In addition to the fact that situated language use is cognitively demanding, there are other reasons to consider the importance of cognitive resources for in-situ language processing. There is a broad body of research in psycholinguistics, neuroscience, and experimental psychology that argues that language cannot be separated from what is called ‘domain general’ cognition (e.g., Hasson et al., 2018; Hagoort, 2016; Pulvermuller & Berthier, 2008; Skipper, 2015; Willems & Hagoort, 2007). Current neuroscientific and neurological models of language propose a distributed network, rather than a few core areas (e.g., Dick et al., 2013; Marebwa et al., 2017; Pulvermuller & Berthier, 2008; Skipper, 2015). For example, the comprehension of narratives activates large areas of the cerebral cortex, and not simply those canonically viewed as ‘language’ areas (Deniz et al., 2019; Huth et al., 2016). Research into embodied cognition has demonstrated that our cognitive representations, such as the meanings of words and sentences, are not abstract, but are composed of rich perceptual information and potential motor actions (Meteyard et al., 2012; Spivey et al., 2009). In parallel, the ability to trace white matter has highlighted that the integrity of white matter connections across such a network are important for preserved language processing post-stroke (Marebwa et al., 2017), and damage to white matter may in fact be a more important contributor to the long-term recovery of language skills than damage to grey matter (Gajardo-Vidal et al., 2021). Damage to a network means that it is less efficient, has less redundancy, and is less able to transmit information successfully.

Considering language as a distributed association network (e.g., Varley, 2011), is an interesting and long-standing hypothesis that suggests that impairments in aphasia are caused by bottlenecks or reductions in the cognitive resources available during language processing (e.g., Dick et al., 2001; van Ewijk & Avrutin, 2016). For example, inefficient allocation of attention during language processing explains the finding that slower presentation, larger fonts, and other scaffolding can produce correct responses from people with aphasia who previously made errors (McNeil et al., 1991). A reduction in processing capacity explains why people with aphasia struggle with tasks that have increased linguistic complexity (e.g., syntactic complexity) or with tasks that are more complex (e.g., dual-tasks; Caplan & Hildebrandt, 1988; Caplan, 2012; Miyake et al., 1994). Recently, processing speed has been shown to be a strong predictor of functional outcomes for people with traumatic brain injury along with learning, memory, and attention (Wilson et al., 2020). For people with aphasia, slow or delayed processing has been demonstrated for sentence comprehension (Haarmann & Kolk, 1991), word retrieval (Faroqi-Shah & Gehman, 2021), and problems with timing, that is, a delay in bringing together different cognitive-linguistic processes that are required for communication (Kolk, 2006; Avrutin, 2000). Such issues would certainly lead to difficulties in the dynamic setting of real-world communication, where a multitude of processes occur simultaneously. Finally, there is evidence for executive dysfunction in aphasia that impairs the selection of context appropriate, goal-directed language (e.g., Almaghyuli et al., 2012; Thompson et al., 2018). In sum, the integration and use of multiple channels of information during comprehension may be associated with the processing capacity that is available (i.e., resource limitations) or the ability to be flexible in using different signals at different times to aid comprehension (i.e., resource allocation). Similarly, the strategic use of multiple channels of communication during language production for total communication may be linked to executive control and the ability to flexibly select appropriate responses.

There are strong grounds to conclude that part of the impairments in aphasia are due to a reduction in cognitive resources deployed for language processing, such as attention, working memory, and the ability to strategically and flexibly select an appropriate response. As discussed in the current review, part of the challenge of situated language processing for some people with aphasia is the fact that language processing is a more complex situation that places heavier demands on attention, working memory, and flexible responses. For other people with aphasia, situated language processing may be less challenging due to the multitude of facilitatory processes that are present in face-to-face communication (e.g., the conversation partner, multiple channels of information, the context, etc.). Understanding how the linguistic and cognitive impairment profiles of people with aphasia relates to the ability to use the facilitatory factors present in face-to-face communication is a key challenge for future work in the field.

### Clinical Implications of the Current Framework

#### Assessment

Current practice for quantifying everyday communication is that either a large number of heterogeneous instruments are used (Doedens & Meteyard, 2020), or standardised impairment-based aphasia assessment batteries are relied upon as secondary outcome measures (Brady et al., 2016; Verna et al., 2009). We have used the proposed framework to evaluate existing published measures of functional communication, finding that the Scenario Test (van der Meulen et al., 2010) is most comprehensive in its assessment, although it requires some modifications to fully capture communicative competence (Doedens & Meteyard, 2020).

The theoretical framework provides a guide for how researchers should seek to improve clinical assessment. Assessment of functional communication should explicitly measure the interactive, multimodal, and contextual aspects of a person’s communicative abilities. Interactivity means that an assessment instrument should evaluate a person’s ability to communicate with at least one other person. Multimodality means that assessment of functional communication should consider the communicative effectiveness of individual channels of information such as language, gesture, prosody, eye-gaze, writing, and drawing in a communicative exchange, as well as the combined value of these communication channels, both in production as well as comprehension. To account for common ground, a person’s ability to rely on shared and pre-existing knowledge can be evaluated. For example, does an individual communicate more effectively with a familiar person compared to a stranger, and do they communicate more effectively on familiar topics? Does someone benefit from the communicative context by using shorter expressions to refer to something that has already been said throughout the course of the conversation? Finally, assessment should indicate whether someone is able to benefit from using their physical environment. For example, can they spontaneously use physical objects in the environment for reference? Is talking about what to have for dinner easier in the supermarket or the kitchen compared to the living room or the park? A published assessment like the Scenario Test could be used to capture communication on unfamiliar topics with a relatively unfamiliar person, in this case the clinician, but a complete assessment may need to measure the potential benefits of familiar places, people, and topics, for example, when communication takes places in the person’s familiar home environment with familiar conversation partners. This is non-trivial. For example, if a person with aphasia is able to benefit from the familiarity of their conversation partner or topic of conversation, a goal for intervention may be to improve performance with personally relevant, unfamiliar people and topics, towards the level of performance seen with familiar people and topics. In addition, a measured benefit of familiarity can be used to illustrate a communicative strength to the person with aphasia.

The framework delineates three major components, which in turn can be broken up into different sub-components (see Clark, 1996). It is likely that, due to the heterogeneity of aphasia symptoms, these components will be differently impaired in each person (Brady et al., 2016), resulting in a different overall communication profile for each person with aphasia. This profile will, in turn, interact differently across various external factors such as different conversation partners, different settings, and contexts, which may be more or less supportive for communication (Harmon, 2020; Ramsberger & Menn, 2003). The aim for measures of communicative ability should be to compile a profile of skills. Note that this is, in a way, similar to how a number of aphasia batteries currently provide scores across different component linguistic skills.

#### Therapy

The current review suggests that processing linguistic information in the dynamic environment of real-world communication could represent a different cognitive challenge compared to working with linguistic materials in an isolated, controlled environment. For some people with aphasia, the lack of generalisation of therapy effects might be due to their inability to apply the newly trained decontextualized linguistic skills to the dynamics of real-world communication. If we want to see improvements in real-world communication, those skills may have to be targeted directly. This process can be likened to re-learning how to walk. In order to re-learn how to walk, it may not be sufficient to solely rely on a rigorous gym protocol to strengthen the leg muscles. For some people, additional training may be required to retrain the muscles to coordinate their actions to walk, jump, and climb various kinds of stairs and uneven surfaces again. For people with aphasia, the rehabilitation of their communication skills may require training the use of linguistic materials that have been targeted in impairment-based approaches in increasingly complex, communication-like, and increasingly cognitively demanding settings, such as one-to-one conversation and group therapy, to ensure these skills effectively carry over to real-world communication (Bastiaanse & Prins, 1994). Therapeutic interventions could be placed on a continuum, gradually moving from decontextualised to contextualised settings of training. More fundamental research in neurorehabilitation supports this approach. Reviewing work on neuroplasticity, Raymer et al. (2008) point out that “generalisation is most likely to occur to a language behaviour that is similar to the trained language behaviour” (p. S265) and that “greater functional outcomes... are more likely when rehabilitation incorporates complex tasks and/or environments” (p. S263). For some people with aphasia, this may extend as far as having to practice in ‘real’ settings outside the clinic or to construct tasks that mimic those settings.

Exploring ways of incrementally building complexity into the therapeutic setting is part of many speech and language therapists’ daily practice. These kinds of approaches are increasingly being formalised and reported in research (Breitenstein et al., 2017). Studies on aphasia rehabilitation in group settings, for example, often exemplify a hierarchical approach towards generalisation of treatment (Elman & Bernstein-Ellis, 1999; Fama et al., 2016; Hoover et al., 2015; Kagan & Simmons-Mackie, 2007; Stahl et al., 2016; ). Other examples include studies that assess the treatment of one linguistic level, such as word retrieval, integrated into the context of a higher linguistic level, such as at sentence or at discourse level, with the aim of facilitating generalisation of therapy effects into everyday communication (Boyle, 2011; Herbert et al., 2003; Murray et al., 2007; Raymer et al., 2006; Webster & Whitworth, 2012). It is rare, however, to find studies that extend the therapeutic intervention to the level of dynamic, interactive, multimodal exchanges as described by the current framework. Most intensive comprehensive aphasia programs (ICAP) combine decontextualized individual and computer treatment with interactive group therapies and functional communication therapy (Breitenstein et al., 2017; Hoover et al., 2017; Rose et al., 2013a). There is an increasing variety of ICAP programs (Rose et al., 2013a) defined in terms of intensity and the components of the WHO ICF targeted (impairment, activity, participation, and wellbeing; World Health Organization, 2001). As these therapy programs include multiple different interventions simultaneously, it can become difficult to discern which therapy or therapeutic mechanisms contribute to improvements made at the level of functional communication, if improvements are found. Put differently, there is a risk that such approaches may lose sight of the critical elements of therapy, or the therapeutic mechanisms, that produce gains in functional communication. Finally, there are many different ’conversational’ therapies that directly target skills at the level of everyday communication. Surprisingly few (less than 20%) focus on training the conversational skills of the person with aphasia themselves (Simmons-Mackie et al., 2014).

Our argument is that, given the importance of functional communication, efforts should be focused on therapies that explicitly incorporate interactive, multimodal, and contextually driven therapy protocols. The framework provides therapists with a structure to specifically plan their interventions according to the different task demands and components of situated language use. Interactivity means that therapy must involve at least one other person with whom goal driven communicative tasks are taking place. Multimodality means that the therapy employs multiple channels of communication such as speech, eye-gaze, prosody, gesture, writing, or drawing in both production and comprehension. Common ground and contextually driven means that there is shared understanding and a shared goal for communication between the interlocutors. It also means that the physical environment is taken into account when conducting a therapy session, either by creating a more naturalistic setting in the clinic room by using, for example, physical props such as objects to be discussed or pictures of scenes, or by varying the location in which communicative tasks are taking place. Examples of different physical environments include places with more distractions such as a waiting room or a local café. As long as the communicative task takes place between two people and lasts for more than a few exchanges, communicative context will automatically be built. The degree to which the conversation partners know each other, and the degree to which the conversation partner is aware of the goals of the task, can be varied in order to manipulate common ground. People with aphasia often report greater difficulty communicating with strangers or on unfamiliar topics, making this a potential target for therapy (e.g., Ferguson, 1994; Green, 1982; Howe et al., 2008, Laakso, 2014b). Such a goal may be approached by playing a simple communication game that includes unfamiliar topics, or may be complex, such as a prolonged conversation on an abstract topic.

It is interesting to note that paired or group settings will almost immediately meet all the criteria for functional communication therapy. An example is the original design of Promoting Aphasic Communicative Effectiveness (PACE) therapy, or variations of this, as described by Davis (2005) and Pulvermüller and Roth (1991). Language games are also increasingly used as the basis for group therapies and can be adapted depending on the goal of the intervention and the severity of the aphasia. The goal of such interventions may vary from word-finding (e.g., Romani et al., 2018; Stahl et al., 2016) to effective switching between different channels of information such as gesture and drawing (e.g., Purdy & Wallace, 2015), to performing speech acts as described by Pulvermüller and Roth (1991), to storytelling (e.g., Carragher et al., 2020). Thus, these methods can be used to target specific communicative therapy goals, and to extend and incorporate “traditional language stimulation techniques into a communicatively dynamic context” (Peach, 2001, p506, as quoted by Davis, 2005). An example of a word-retrieval treatment according to the highly structured ‘core’ PACE principles (Davis, 2005) is as follows. One person is required to describe an item on a card to a conversation partner, who has not seen the card. The conversation partner may ask questions to clarify, creating a back-and-forth dialogue that is more like real-world settings than a traditional picture description task. The variations in such a setup are virtually endless. The items presented on the cards can be tailored to elicit previously trained target words, and can vary in complexity by changing single objects to more complex images of scenes, or by using multiple cards that differ on specific characteristics, for example, on colour or shape (Pulvermüller & Roth, 1991). Games can be modified to include a greater use of multimodal communication to support the strategic use of these other communication channels (e.g., Purdy & Wallace, 2015). The degree of shared common ground can be varied by manipulating how much shared knowledge the conversation partner has. For instance, the partner may have a set of options in front of them from which to choose the card (high shared knowledge or common ground), they may only have cues about the category (medium shared knowledge), or they may not know what is going to be described, similar to many situations in everyday life (low shared knowledge). This could also be titrated during the intervention. Finally, the interactivity between the interlocutors can be varied by relying on different conversation partners for the game, who have different ways of responding and vary in their ability to support communication flexibly. Whilst traditionally the role of the clinician, friends, family members, other people with aphasia, or volunteers can take on the role of conversation partner to examine and improve the ability of the person with aphasia to adapt effectively to different responses, different levels of communicative support, and levels of understanding.

In simple terms, the end stages of intervention should include tasks that mimic, or seek to mimic, real-world situations in some form, beginning with the selection of personally relevant events, topics, and situations (Barnes & Bloch, 2018; Byng et al., 2013; Haley et al., 2019; Hersh et al., 2012). Anecdotal examples from our own work include a photographer practising phrases to organise groups during wedding shoots, a gentleman who wanted to independently buy his lottery ticket every week and start conversations at a hobby group, and a mother and daughter who wanted to improve their daily conversations. From that point, tasks can be developed that ideally build up to the target situation, such as practise buying the lottery ticket, or similar such as role play to practice buying the lottery ticket. Impairment based, in vacuo tasks fit naturally into a hierarchy of practising the production or comprehension of target words and phrases and may be the starting point for intervention.

Similarly, the framework makes it possible to break down the components associated with different goals for improving communication. For example, a goal of placing a bet in a betting shop and filling out a betting slip, will have a different situational and interactive contexts, and different task demands compared to a goal of placing a bet online, using an iPad. We would like to note here that in our clinical work and in informal conversations, speech and language therapists often report working in the aforementioned manner by targeting everyday communicative behaviours. However, in our opinion, the research literature does not yet adequately reflect the way in which clinical work can, and should, systematically target functional communication. Potential next steps for research lie in the systematic investigation of interactive, multimodal, and contextualised interventions, and the potential of these interventions to improve specific communicative behaviours in everyday life, either directly, or as a way to promote generalisation from more impairment-based interventions. One hypothesis is that structured interventions that include these features will show better generalisation to everyday communication than those that do not. We note that the movement towards ICAP models of intervention is a strong argument for highly structured, resource intensive intervention packages that are time limited (Rose et al., 2013b). New developments in technology may also provide possibilities for this (e.g., Carragher et al., 2020; Bryant et al., 2019). The tasks we have described above would fit with such models.

Finally, as stated in the previous section on *Cognition*, there is a growing body of evidence that suggests that difficulties in aphasia relate to limitations in the attention, working memory, and executive resources available for language and communication. These cognitive resources may improve naturally as part of the implementation of more situated, naturalistic tasks (e.g., Cherney & Halper, 2008), or enhanced by beginning with impairment-based repetition and drills to make particular words, phrases, or sentences more salient and automatically available (Bilda, 2010; Meteyard et al., 2014). Another way to approach this in rehabilitation is through meta-cognitive training. That is, training people with aphasia to understand real-world communication as a skill set, to reflect on their own skills, and to implement strategies to improve this skill set. For example, consciously switching between different modalities or channels of information (e.g., Olsson et al., 2019; Purdy & Wallace, 2015), monitoring for feedback during interactive communication (e.g., similar to Beeke et al., 2011) or understanding and adapting to the way in which limited working memory and attentional capacity affects communication, and adapting communication accordingly (e.g., Ruiter et al., 2010; Springer et al., 2000). Finally, these cognitive resources might be targeted directly through therapy, with the aim of improving functional communication (e.g., Adjei-Nicol, 2020; Murray, 2012; Peach et al., 2017; Ramsberger, 2005; Salis et al., 2017).

## Conclusion

We have presented a systematic and theoretically founded framework of real-world communication. The framework provides a delineated set of principles that underpin language use in the real world. This framework provides clear steps for future research to systematically investigate real-world communication in people with aphasia. It is of crucial importance for the development of effective assessment and interventions in aphasia rehabilitation to have a thorough understanding of what communication is, what skills are required to communicate in the real world, and how the behaviours targeted in therapy can be generalised to real-world language use. These insights are needed to better understand the discrepancies between linguistic scores and real-world communicative abilities in people with aphasia across impairment profiles. The authors hope this paper will illustrate and emphasize the importance of studying, assessing, and treating *communication* as a behaviour that is different from *language* as a solely linguistic phenomenon, and that working at the level of *communication* requires taking into account the different task demands and resources that may be used to communicate effectively.

## Footnotes

1 The ability to communicate in the real world or in one’s own everyday life will be referred to as functional communication or simply as communication. This refers to skills, including language skills, required to communicate in various situations one might come across in one’s day-to-day life.

2 The term *generalisation* can refer to different types of change after therapeutic intervention, as described by Webster, Whitworth & Morris (2015). We will use this term to mean an improvement in behavioural performance or an improvement on an assessment measure that was not directly targeted by a therapy task.
